# Beyond adaptive cruise control and lane centering control: drivers’ mental model of and trust in emerging ADAS technologies

**DOI:** 10.3389/fpsyg.2023.1236062

**Published:** 2023-08-08

**Authors:** Chunxi Huang, Dengbo He, Xiao Wen, Song Yan

**Affiliations:** ^1^Robotics and Autonomous Systems, Division of Emerging Interdisciplinary Areas (EMIA) under Inter-disciplinary Programs Office (IPO), The Hong Kong University of Science and Technology, Kowloon, Hong Kong SAR, China; ^2^Intelligent Transportation Thrust, The Hong Kong University of Science and Technology (Guangzhou), Guangzhou, China; ^3^HKUST Shenzhen-Hong Kong Collaborative Innovation Research Institute, Futian, Shenzhen, China; ^4^Department of Civil and Environmental Engineering, The Hong Kong University of Science and Technology, Kowloon, Hong Kong SAR, China

**Keywords:** mental model, trust, ADAS, survey study, cluster analysis

## Abstract

**Introduction:**

The potential safety benefits of advanced driver assistance systems (ADAS) highly rely on drivers’ appropriate mental models of and trust in ADAS. Current research mainly focused on drivers’ mental model of adaptive cruise control (ACC) and lane centering control (LCC), but rarely investigated drivers’ understanding of emerging driving automation functions beyond ACC and LCC.

**Methods:**

To address this research gap, 287 valid responses from ADAS users in the Chinese market, were collected in a survey study targeted toward state-of-the-art ADAS (e.g., autopilot in Tesla). Through cluster analysis, drivers were clustered into four groups based on their knowledge of traditional ACC and LCC functions, knowledge of functions beyond ACC and LCC, and knowledge of ADAS limitations. Predictors of driver grouping were analyzed, and we further modeled drivers’ trust in ADAS.

**Results:**

Drivers in general had weak knowledge of LCC functions and functions beyond ACC and LCC, and only 27 (9%) of respondents had a relatively strong mental model of ACC and LCC. At the same time, years of licensure, weekly driving distance, ADAS familiarity, driving style (i.e., planning), and personability (i.e., agreeableness) were associated with drivers’ mental model of ADAS. Further, it was found that the mental model of ADAS, vehicle brand, and drivers’ age, ADAS experience, driving style (i.e., focus), and personality (i.e., emotional stability) were significant predictors of drivers’ trust in ADAS.

**Discussion:**

These findings provide valuable insights for the design of driver education and training programs to improve driving safety with ADAS.

## 1. Introduction

Advanced driver assistance systems (ADAS) have developed rapidly and have become increasingly prevalent worldwide over the past decade (Winter et al., 2014; Banks and Stanton, 2016; Litman, 2020). Typically, ADAS in SAE Level 2 level (SAE International, 2021) can provide vehicle control in the longitudinal direction with adaptive cruise control (ACC) or cruise control systems, and in the lateral direction with lane keeping assistance (LKA) or lane centering control (LCC) systems. With the advancement in hardware (e.g., sensing technologies) and software (e.g., computer vision algorithms) in recent years, new ADAS functions beyond ACC and LCC (e.g., automated lane changing, and automated overtaking) are being integrated into vehicles. Further, instead of introducing each ADAS function as an independent system, vehicle companies tend to “pack” all ADAS functions as a single system. For example, Tesla names its ADAS as Navigate on Autopilot (NOA) (Tesla, 2020) and XPeng names its ADAS as Navigation Guided Pilot (NGP) (XPeng Motors, 2021), both include all functions of ACC and LCC, but can also functions beyond ACC and LCC. To differentiate the driving automation systems such as NOA and NGP from traditional SAE Level 2 ADAS that consists of ACC and LCC, throughout this paper, we will name driving automation systems such as NOA and NGP as advanced Level 2 ADAS.

However, it should be noted that regardless of how vehicle manufacturers name them, users of these advanced Level 2 ADAS (i.e., NOA and NGP) are still expected to be responsible for the driving task and be ready to quickly take over control of the vehicle in case of emergency. Thus, users’ knowledge of and trust in ADAS may still play a critical role in driving safety. To systematically evaluate users’ knowledge of ADAS, previous studies used the concept of ADAS mental model and defined it as drivers’ understanding of the functions, limitations, and capabilities of ADAS (e.g., Beggiato and Krems, 2013; Beggiato et al., 2015; Rossi et al., 2020; Pai et al., 2023). However, to the best of our knowledge, most existing research mainly focused on traditional ADAS technologies (e.g., Beggiato and Krems, 2013; Beggiato et al., 2015; DeGuzman and Donmez, 2021a,b; Lubkowski et al., 2021). Given that even experienced ADAS users have difficulty understanding all capabilities and limitations of ACC and LCC (DeGuzman and Donmez, 2021b), it may be more challenging for drivers to build well-calibrated mental models of advanced Level-2 ADAS, especially the functions and limitations of the sub-systems beyond ACC and LCC.

The misuse of the ADAS has contributed to a large number of accidents in the past few years (National Highway Traffic Safety Administration, 2022). Considering the increasing market share of advanced Level-2 ADAS, to ensure drivers’ proper use of the state-of-the-art ADAS technologies, it becomes urgent to understand how well drivers know about ADAS and what factors influence drivers’ knowledge of ADAS, which could help calibrate users’ trust in these systems and avoid overuse or misuse of them. More importantly, according to the data collected by the Ministry of Industry and Information Technology in China (Ministry of Industry and Information Technology, 2022), in the first six months of 2022, more than 2.88 million passenger cars with ADAS were sold in China (accounting for 32.4% of all vehicles sold in that period) and with a year-on-year growth of 46.2% (NetEase Auto, 2022). However, no research has been conducted among Chinese drivers to explore their mental models of and trust in ADAS. Due to the potential cultural differences between Chinese drivers and drivers with other culture background, it is important to investigate Chinses drivers’ mental models of and trust in ADAS, especially considering the rapidly increasing of ADAS penetration rate in China.

## 2. Background

### 2.1. Mental model of ADAS is critical to driving safety

To secure the benefits of ADAS, drivers need to have an appropriate understanding of how, when, and whether ADAS can be used. Specifically, drivers’ mental models of traditional Level-2 ADAS (i.e., knowledge of the functions and limitations of the ADAS) were found to be associated with driving safety, when either the driving automation was responsible for the longitudinal control (i.e., through ACC) or lateral control of the vehicles (i.e., through LCC) (Dickie and Boyle, 2009; Rossi et al., 2020; Gaspar et al., 2021).

In general, previous studies revealed that drivers who are less aware of the functions and limitations of traditional Level-2 automation (e.g., ACC and LCC) are more likely to fail regaining control of the vehicle when needed (Solís-Marcos et al., 2018). For example, in a driving simulator study, Gaspar et al. found that drivers with strong mental models of ACC were faster in responding to edge-case situations (i.e., when ACC failed to detect an approaching object in front) compared to those with weak mental models of ACC (Gaspar et al., 2021). In another study, Dickie and Boyle found that drivers who were unaware or unsure of ACC limitations were more willing to use the automation in situations that were beyond the system’s capabilities (Dickie and Boyle, 2009). At the same time, drivers who were unaware of ACC and LCC limitations have been shown to attend less to the road (Noble et al., 2021), and be more inclined to perform non-driving related tasks (Carsten et al., 2012; Noble et al., 2021), and thus be less prepared to intervene when they need to (Winter et al., 2014), which may impair traffic safety (Gold et al., 2018; Wan and Changxu, 2018). As for the lateral control of the vehicle, through a driving simulator study, Rossi et al. (2020) found that in takeover scenarios, drivers’ mental model of ADAS was a significant predictor of takeover effectiveness, as measured by the mean absolute lateral position and standard deviation (SD) of the lateral position of the vehicle. In the study, those who have ever read the information booklet of ADAS (i.e., the stronger mental model drivers) exhibited smaller SD of lateral position and responded faster in the events. Thus, it is necessary to help drivers construct a well-calibrated mental model to ensure safety benefits of ADAS.

### 2.2. Mental model can affect trust in ADAS

The relationship between the mental model and trust may explain the influence of the mental model on driving safety. Trust in automation can be defined as “*the attitude that an agent will help achieve an individual’s goals in a situation characterized by uncertainty and vulnerability.”* (p. 54 of Lee and See, 2004). Previous research has found that users’ level of trust in ADAS has been associated with their performance in perceiving the environment (Radlmayr et al., 2014), reacting to takeover events (Hergeth et al., 2016; Payre et al., 2016), and responding to hazards (Seppelt and Lee, 2007).

Drivers’ mental model of ADAS can influence their trust in the ADAS (Lee and See, 2004; Hoff and Bashir, 2015). For example, in a survey study conducted in North America, DeGuzman and Donmez found that among ADAS non-owners, those with a better mental model of the ADAS reported lower trust toward the ADAS (i.e., ACC and LKA) (DeGuzman and Donmez, 2021b). In another survey study, drivers who were unaware or unsure of ACC limitations were found to be more willing to use the automation in situations that were beyond the system’s capabilities (Dickie and Boyle, 2009), indicating these drivers may have over-trusted the ADAS they used.

It should be noted that drivers’ trust in ADAS is not solely determined by the mental model of the system but can also be moderated by other factors. According to the framework by Hoff and Bashir (2015), there are “*three layers of variability in human–automation trust*,” i.e., dispositional trust (i.e., an individual’s overall tendency to trust automation, independent of context or a specific system), situational trust (i.e., transitory context characteristics, types of the system, its complexity and the difficulty of the tasks), and learned trust (i.e., an operators’ evaluation of the systems learned from past experience or current interactions). Each layer of variability can be modulated by different factors. For example, dispositional trust might be associated with age, gender, culture, personality traits; situational trust can be influenced by system complexity and workload; and learned trust can be associated with users’ experience with and understanding of the system, as well as their mental of the system. All the factors associated with different layers of trust can influence users’ trust in automation directly or indirectly. For example, previous research found that factors from dispositional layer (e.g., age and education) (Gold et al., 2015), situational layer (e.g., traffic density) (Li et al., 2019) and learned layer (e.g., consistency of driver’s driving style and the driving style of driving automation) (Ma and Zhang, 2020, 2021) can influence drivers’ trust in ADAS. Thus, it is necessary to consider other potential moderating factors when investigating the relationships between the mental model and drivers’ trust in ADAS.

### 2.3. ADAS users have weak mental model of ADAS

Though the mental model of ADAS is critical to driving safety, research has found that drivers generally have less than ideal mental models of traditional Level-2 ADAS in general. For instance, in a survey study, Dickie and Boyle (2009) found that only 42% of ACC owners were aware of the three ACC limitations mentioned in the survey. Similarly, Jenness et al. (2008) found that 72% of ACC owners were not aware of some critical limitations of the ACC. In another survey study targeted toward Volvo XC60 (equipped with ACC system) owners, around 30% of the 130 respondents reported being unaware that the system has difficulty functioning in curves and roundabouts (Larsson, 2012). What is more alerting is that, though ACC has been available in the market for decades, a large number of drivers still have an incorrect perception of it according to more recent studies. For example, in a survey conducted in the US, McDonald et al. (2016) found that only 17% of the respondents correctly answered questions assessing their understanding of the ACC system. A more recent study by DeGuzman and Donmez (2021a) still found that 47% of owners hold misperceptions about the ACC systems (e.g., incorrectly thought that ACC systems would not have difficulty when driving on curvy roads or when approaching a stationary vehicle).

Not just for ACC, drivers also have difficulty understanding other driving automation functions. In another survey study with over 1,500 drivers, Jenness et al. (2007) found that 81% of respondents were unaware of any limitations of the sensor-based backing aid system, believing that the system was designed to detect the proximity of pedestrians, children, pets, and stationary obstacles, which it was not designed to. Similarly, A driving simulator study found that, among 24 drivers with a basic understanding of the lane departure warning (LDW) system, 20 of them mistakenly believed that the system would work at any speed (Aziz et al., 2013).

It is alerting that drivers have weak knowledge of ADAS functions that have been in the market for many years. The more recent and complex ADAS functions introduced in the past few years may bring even more challenges to drivers. Thus, it is urgent to assess drivers’ mental models of advanced Level-2 ADAS.

### 2.4. Current study

To address the research gap, a survey study has been conducted to evaluate drivers’ mental models of advanced Level-2 ADAS and further explore the relationship among the mental model of ADAS, trust in ADAS as well as other potentially underlying factors. According to the driving task model by Ranney (1994), traditional ADAS technologies (e.g., ACC and LCC) help drivers perform the driving task at the operational level and the judgment of the system performance can solely be based on the perception of the current maneuver of the system. While emerging ADAS technologies (e.g., automatic lane changing) can help drivers perform the driving task at the tactical level. To judge the performance of and develop trust in these systems, drivers will need a higher level of situation awareness of the traffic scenario. The discrepancy of the required cognitive resource to judge the system performance may affect how the mental model and trust are built up and evolve when using traditional versus advanced ADAS. At the same time, given the expanding market share of advanced Level-2 ADAS and the increasing complexity of this system, existing research focusing on traditional ADAS functions (i.e., ACC, CC, LKA, or LCC) may not be enough to support appropriate usage of the emerging technologies in vehicles. Further, the traditional functions in ADAS have been available in the market for an extended period and it is valuable to compare drivers’ mental model of these emerging functions versus the mental models of functions that drivers are potentially more familiar with. This comparison can help better understand the impact of new vehicles technologies on the driving safety. Thus, our survey study targeted the functions and limitations of advanced Level-2 ADAS functions in addition to the traditional Level-2 ADAS functions.

Further, cultural difference has been identified as a potentially influential factor in users’ trust in automation (Hoff and Bashir, 2015). Previous studies on drivers’ mental model as well as trust toward driving automation mainly targeted toward users in North America (e.g., DeGuzman and Donmez, 2021a; Greenwood et al., 2022; Hungund and Pradhan, 2022) or Europe (e.g., Larsson, 2012; Beggiato and Krems, 2013; Beggiato et al., 2015). Given the rapidly increasing market share of advanced Level-2 automation in China (Ministry of Industry and Information Technology, 2022), the increasing number of ADAS-related accidents in China (Autoevolution, 2021), and the potential cultural difference in Asia (Zaheer and Zaheer, 2006), understanding of Chinese drivers’ mental model of ADAS may provide valuable insights on the design of customized in-vehicle interfaces and driver education programs.

For the current study, we hypothesized that, compared to emerging ADAS technologies, drivers have better mental models of traditional ADAS technologies, given that understanding and judging emerging ADAS technologies require more cognitive resources compared to that of traditional ADAS technologies. Inspired by previous studies (Beggiato and Krems, 2013; Beggiato et al., 2015; DeGuzman and Donmez, 2021a,b; Lubkowski et al., 2021; Greenwood et al., 2022), we also explored other potential related factors and we expect that higher ADAS familiarity, higher technology familiarity, more ADAS experience, and higher ADAS frequency are positively associated with better ADAS mental models, while involving in ADAS-related accident is negatively associated with drivers’ trust in ADAS. As for the relationship between drivers’ mental models of ADAS and trust in ADAS, in general, we hypothesize that better mental model is associated with lower trust in ADAS (Pai et al., 2023), but this trend can be modulated by other factors. For example, previous research found that the experience of using ADAS might weaken the influence of mental model on trust (DeGuzman and Donmez, 2021a).

## 3. Materials and methods

### 3.1. Instrument

As shown in Table 1, the questionnaire used for the survey study included three parts: (1) demographics and driving-related information; (2) assessment of drivers’ knowledge of and trust in ADAS; (3) and assessment of drivers’ driving styles and personalities.

**Table 1:** Questions in the survey, extracted variables, and the distribution of the variable.

Description	Variable^[Type]^	Distributions
The age (in years of old) of the participant.	Age^[C]^	M = 29.9 (SD: 6.1) Min: 20, Max: 58
The gender of the participant	Gender^[N]^	Male (*n* = 262) Female (*n* = 25)
The highest education level of the participant.	Education^[N]^	Professional college or less (*n* = 86) Bachelor or above (*n* = 201)
The working status of the participant.	Working status^[N]^	Not work (*n* = 10) Self-employed (*n* = 115) Full-time work (*n* = 153) Others (*n* = 9)
The marriage status of the participant.	Marriage status^[N]^	Married (*n* = 173) Not married (*n* = 114)
The household income of the participant (in RMB).	Household income^[N]^	Less than 140,000 (*n* = 35) 140,000–250,000 (*n* = 108) 250,000–700,000 (*n* = 112) 700,000 - 1,000,000 (*n* = 23) Over 1,000,000 (*n* = 9)
Participant’s self-reported familiarity with technology.	Technology familiarity^[C]^	M = 7.2 (SD: 1.3) Min: 4.3, Max: 10
The brand of the vehicle that the participant owned.	Vehicle brand^[N]^	Tesla (*n* = 141) Others (*n* = 146)
The duration of possession of the current vehicle.	Possession period^[N]^	Within 1 year (*n* = 95) Over 1 year (*n* = 192)
For many years since participants obtained their first driver’s license.	Years of licensure^[C]^	M = 7.4 (SD: 4.4) Min: 1, Max: 28
Participant’s self-reported driving frequency in the past year.	Driving frequency^[N]^	Almost every day (*n* = 148) Several times per week (*n* = 107) Several times per month (*n* = 30) Several times per year (*n* = 2)
Participant’s self-reported weekly average driving distance in the past year.	Weekly driving distance^[N]^	Less than 99 km (*n* = 122) 100–299 km (*n* = 106) Over 300 km (*n* = 59)
Participant’s self-reported experience of ADAS.	ADAS experience^[N]^	Less than 1 month (*n* = 23) 1–6 months (*n* = 127) 6–12 months (*n* = 85) Over 12 months (*n* = 52)
Participant’s self-reported frequency of using ADAS.	ADAS frequency^[N]^	Almost every day (*n* = 50) Several times per week (*n* = 138) Several times per month (*n* = 79) Several times per year (*n* = 18) Almost no (*n* = 2)
Participant’s self-reported accident history with ADAS.	ADAS accident history^[N]^	Yes (n = 27) No (*n* = 260)
Participant’s self-reported familiarity with ADAS.	ADAS familiarity^[C]^	M = 8.2 (SD: 1.3) Min: 4.3, Max: 10
Participant’s mental model of ADAS, measured by agreement on 49 ADAS-related statements.	OMMS^[C]^	M = 60.4 (SD: 5.7) Min: 44.5, Max: 75.9
	gbMMS-ACC^[C]^	M = 68.4 (SD: 9.6) Min: 38.2, Max: 94.6
	gbMMS-LCC^[C]^	M = 56.2 (SD: 8.7) Min: 37.8, Max: 91.1
	gbMMS-beyond^[C]^	M = 56.0 (SD: 5.4) Min: 36.3, Max: 68.8
	gbMMS-limitation^[C]^	M = 62.2 (SD: 11.9) Min: 15.4, Max: 92.3
Participants’ trust in ADAS.	Trust score^[C]^	M = 4.1 (SD: 0.7) Min: 2, Max: 5
Questions from the ten-item personality (TIPI) measure questionnaire.	Extraversion ^[C]^	M = 4.4 (SD: 1.1) Min: 1, Max: 7
	Agreeableness^[C]^	M = 5.0 (SD: 1.2) Min: 2, Max: 7
	Conscientiousness^[C]^	M = 5.1 (SD: 1.3) Min: 1.5, Max: 7
	Emotional stability^[C]^	M = 5.0 (SD: 1.3) Min: 1, Max: 7
	Openness to experience^[C]^	M = 4.8 (SD: 1.1) Min: 1, Max: 7
Questions from the driving styles questionnaire (DSQ).	Speed^[C]^	M = 8.5 (SD: 4.3) Min: 3, Max: 18
	Calmness^[C]^	M = 11.8 (SD: 3.0) Min: 3, Max: 18
	Social resistance^[C]^	M = 5.9 (SD: 2.3) Min: 2, Max: 12
	Focus^[C]^	M = 13.1 (SD: 2.9) Min: 3, Max: 18
	Planning^[C]^	M = 9.0 (SD: 2.2) Min: 4, Max: 12
	Deviance^[C]^	M = 5.7 (SD: 3.0) Min: 2, Max: 12

C represents the continuous variable; N represents the nominal variable. M stands for mean and SD stands for Standard Deviation.

#### 3.1.1. Part 1: demographics and driving-related information

Previous studies revealed that demographics and driving experience may influence drivers’ acceptance and trust of ADAS (Beggiato et al., 2015; Gold et al., 2015; Dikmen and Burns, 2017; Lee et al., 2019). Therefore, participants’ demographics and driving-related information were collected to explore their potential influence on drivers’ mental models of and trust in ADAS. As shown in Table 1, the demographic information included age, gender, education level, working status, marriage status, household income, and self-reported technology familiarity. The driving-related information included vehicle-related information, driving-experience-related information, and ADAS-experience-related information. Drivers’ ADAS accident history was assessed by one question (i.e., “*Have you ever had an accident when using the ADAS in your vehicle?*”), with possible responses of “*Yes*” and “*No*.” Drivers’ ADAS frequency was assessed using the question “*Please rate your frequency of using ADAS in the past one year*,” with possible responses ranging from “*Almost no*” to “*Almost every day*.” Following previous studies (Chen and Donmez, 2016; DeGuzman and Donmez, 2021a), participants’ self-reported technology and ADAS familiarity were assessed using three questions, i.e., (1) “*the level of experience with technology/ADAS*” with a possible response from 1 (“*very inexperienced*”) to 10 (“*very experienced*”); (2) “*the degree to which participants consider themselves early adopters of technology/ADAS*” with a possible response from 1 (*“absolutely no*”) to 10 (“*absolutely yes*”); (3) and “*how easy they find it to learn new technology/ADAS*” with a possible response from 1 (“*very difficult*”) to 10 (“*very easy*”).

#### 3.1.2. Part 2: assessment of drivers’ mental model of and trust in ADAS

In total, 49 statements were designed to assess drivers’ mental model of ADAS based on a review of previous relevant studies (Beggiato and Krems, 2013; Beggiato et al., 2015; Abraham et al., 2017; McDonald et al., 2018; DeGuzman and Donmez, 2021a,b) and a thorough review of user manuals and official training materials from vehicle manufacturers. The statements targeted toward functions and limitations of traditional Level-2 ADAS and advanced Level-2 ADAS systems and can be categorized into four parts, each targeting toward one type of ADAS-related knowledge, i.e., ACC-related functions, LCC-related functions, functions beyond ACC & LCC, and ADAS limitations.

The ACC-related functions and LCC-related functions parts included statements of what ACC can do (e.g., “*when you drive with ADAS on, the system can help you maintain a pre-set speed*”) and LCC can do (e.g., “*when you drive with ADAS on, the system can help maintain the vehicle in the center of the lane*”). The functions beyond ACC & LCC part assessed participants’ understanding of what the sub-systems beyond ACC and LCC can do. The “beyond” sub-systems include flexible speed control (which can adjust the speed based on road environment such as curvature of the road), automatic stop-and-go (which can continuously follow the lead vehicle in a traffic jam), automatic lane changing (which can automatically change lanes when users turn on turning light or when the lead vehicle is too slow), road assistant (which can identify traffic lights and traffic signs), and driver monitoring & warning (which can monitor drivers’ behaviors during driving and initiate warnings when necessary). The ADAS-limitations group included statements regarding the limitations and boundaries of the ADAS sub-systems (e.g., “*when you drive with ADAS on, the system may have difficulty when driving through construction zones*”).

It should be also noted that for each statement, participants were not informed of the targeted ADAS sub-systems and the names of the sub-systems (e.g., ACC or LCC) were not used in the survey either. Instead, the term “ADAS” was used throughout the questionnaire. This is because drivers may not be aware of the sub-systems but perceive the ADAS as a single system considering this is how vehicle manufacturers introduce ADAS to consumers. Further, we did not classify the limitations into the individual group as some sub-systems share similar limitations, and thus it would be arbitrary to assign the limitations to each sub-system (e.g., bad weather can affect both ACC and LCC). For specific statements (23 out of 49), real-world photos or videos of the scenarios were provided to facilitate understanding of the situations. Instead of responding ‘yes’ or ‘no’, participants were asked to rate their level of agreement with the statement, ranging from 1 (“*strongly disagree*”) to 6 (“*strongly agree*”). Participants were further informed that they should choose ratings below or equal to 3 if they tended to disagree and choose ratings above or equal to 4 if they tended to agree. The full list of all statements used to assess drivers’ ADAS mental models is provided in the Appendix, and the relevant photos and videos are provided in the Supplementary materials.

For the assessment of drivers’ trust in ADAS, following previous research (DeGuzman and Donmez, 2021a), a five-item scale by Jian et al. (2000) was adopted. Participants were asked to rate their overall agreement with the following five items on a five Likert scale ranging from 1 (“Strongly Disagree”) to 5 (“Strongly Agree”), i.e., “*I can trust the ADAS system*,” “*The ADAS system is reliable*,” “*I am confident in the ADAS system*,” “*I am familiar with the ADAS system*,” and “*The ADAS system is dependable*.” This trust assessed by Jian et al. (2000) might be modulated by the factors from the three-layer framework by Hoff and Bashir (2015).

#### 3.1.3. Part 3: assessment of drivers’ driving styles and personalities

To assess the driving styles of participants, the driving style questionnaire (DSQ) was used, which measures six relevant divisions: speed, calmness, social resistance, focus, planning, and deviance (French et al., 1993; West et al., 1993). Possible responses for each question in DSQ range from 1 (“*very infrequently or never*”) to 6 (“*very frequently or always*”). Following previous studies that assessed drivers’ personality (Monteiro et al., 2018; Hussain et al., 2020; Stinchcombe et al., 2023), a ten-item personality measure (TIPI) was used (Gosling et al., 2003), which was a short personality inventory with high reliability and validity for measuring the Big Five personality traits (McCrae and Costa, 2008). Considering that the survey targeted toward Chinese population, a validated Chinese version of TIPI was utilized (Lu et al., 2020), with possible responses ranging from 1 (“*strongly disagree*”) to 7 (“*strongly agree*”).

### 3.2. Participants

Participants were recruited through online posters in vehicle forums and advertisements among interest groups of car owners on social media in China (e.g., WeChat groups of car owners). In total, 781 participants filled out the questionnaire. To be eligible for the study, participants had to be the owner of a vehicle with advanced Level-2 ADAS and had experience using it. Thus, 160 samples were excluded. To screen out potentially unreliable responses and ensure data quality, we further adopted the attention check and survey completion time check following previous studies (Rahman et al., 2018; Ayoub et al., 2021). The attention check was implemented for all participants in our survey by asking a simple math question (e.g., “*If you are reading carefully, please choose the option that matches the result of 6 minus 1*”) and the lower threshold of completion time was set to 6 min (50% of the average completion time of 11.8 min). Thus, 268 samples were excluded. Finally, 66 samples with mismatching information were further excluded (e.g., an unreasonable combination of vehicle brand and vehicle model, and ADAS Frequency being higher than Driving Frequency) after manual review. As a result, 287 valid responses from Chinese ADAS users were kept for analyses in this study. The detailed sample demographic information (i.e., age, gender, education, working status, marriage status, household income) is provided in Table 1. Respondents of the valid survey sample were compensated with 10 RMB. This study was approved by the Human and Artefacts Research Ethics Committee in HKUST (HREP-2022-0117).

### 3.3. Variable extraction

#### 3.3.1. Mental model score

To evaluate participants’ overall mental model of the ADAS functions, an overall mental model score (OMMS) was calculated based on participants’ responses to 49 ADAS knowledge statements. For each statement, the perfect response was defined as 1 (“strongly disagree”) if the statement itself is wrong and 6 (“strongly agree”) if the statement is correct. Inspired by the Manhattan distance, the OMMS of each participant was calculated as follows:


(1) $$OMMS=\left(\overset{49}{\underset{i=1}{\sum }}\frac{5-\left|R_{i}-P_{i}\right|}{5}\;\right)\frac{100}{49}\;$$

where, $R_{i}$ is the actual response from the participant for the $i^{th}$ statement; $P_{i}$ is the perfect answer to the $i^{th}$ statement. Thus, $\left|R_{i}-P_{i}\right|$ is the distance between the participant’s response and the perfect answer, which can be any integer between [0, 5]. Specifically, for a perfect response to a statement, the distance is 0; while for a fully incorrect response, the distance is 5. Therefore, per Equation (1), the possible range for the OMMS is [0, 100].

In addition to the OMMS, we also calculated the group-based mental model score (gbMMS, ranging from 0 to 100) following the equation below:


(2) $$gbMMS_{m}=\left(\overset{length\left(m\right)}{\underset{n=1}{\sum }}\frac{5-\left|R_{mn}-P_{mn}\right|}{5}\;\right)\frac{100}{length\left(m\right)}\;$$

where, $R_{mn}$ is the actual response from the participant to the $n^{th}$ statement of the $m^{th}$ group; $P_{mn}$ is the perfect answer to the $n^{th}$ item of the $m^{th}$ group; length(m) is the number of statements in the $m^{th}$ group. The possible value for m in this study is 1, 2, 3, and 4, representing ACC-related functions group (gbMMS-ACC), LCC-related functions group (gbMMS-LCC), functions beyond ACC&LCC group (gbMMS-beyond), and ADAS-limitations group (gbMMS-limitation).

#### 3.3.2. Other variables

Participants’ familiarity with the technology in general and ADAS were calculated by averaging their responses to the corresponding questions. At the same time, following previous research (Jian et al., 2000), the overall score of trust in ADAS was calculated as the average ratings of five trust-related questions in the questionnaires. Further, according to Gosling et al. (2003), five personality-related variables were derived from TIPI, i.e., extraversion, agreeableness, conscientiousness, emotional stability, and openness to experiences. As for driving style, six relevant divisions of DSQ were calculated following the method by French et al. (1993). All other extracted variables and their distributions can be found in Table 1.

### 3.4. Data analysis

**Figure 1:**
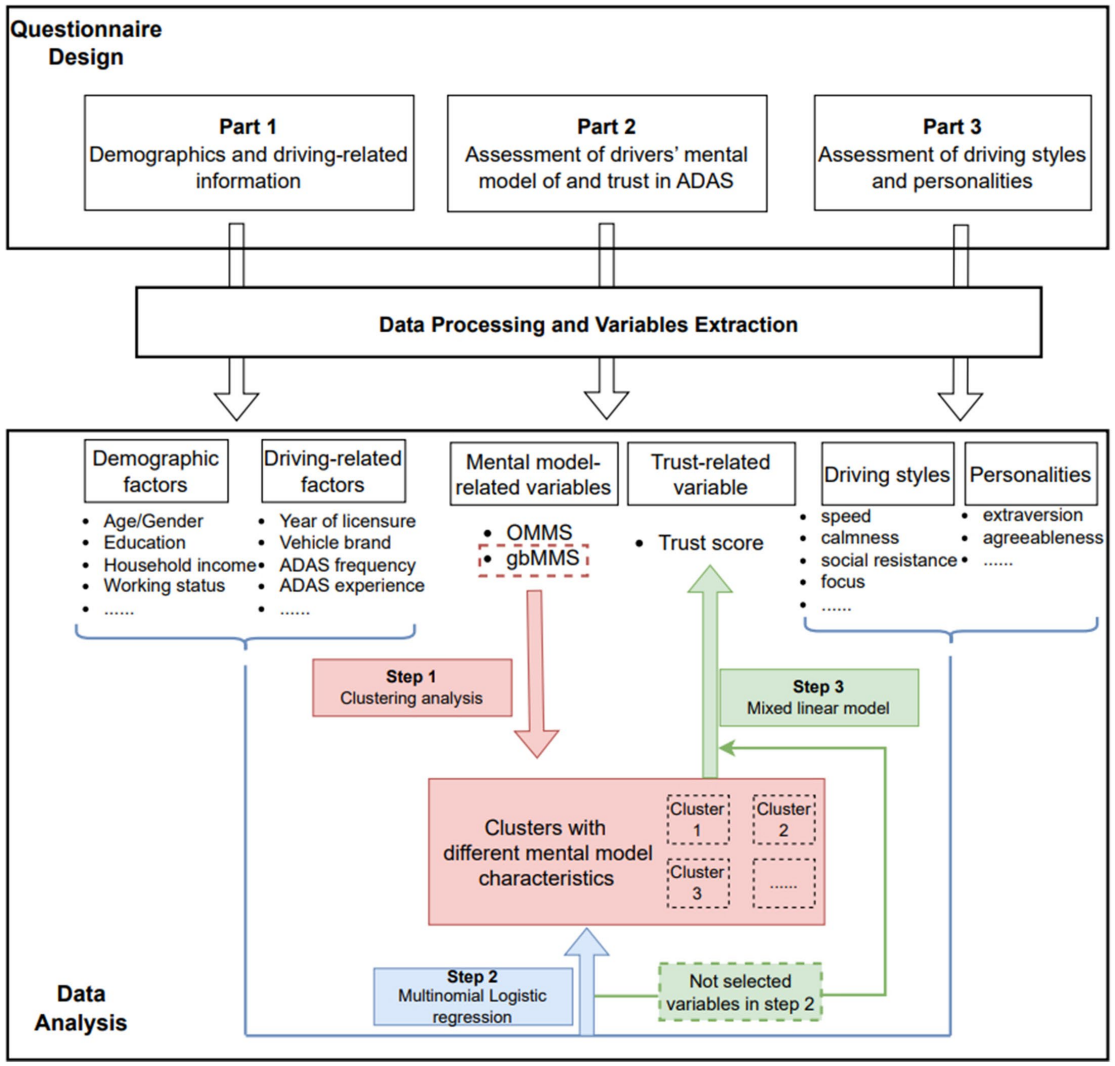
The overall methodological framework of this study.

Figure 1 summarizes the overall methodological framework of our study. Overall, the analyses in this study include three steps: (1) identify driver groups with different characteristics of ADAS mental model through clustering analysis (highlighted in red); (2) explore predictors of drivers’ mental model through logistic regression model (highlighted in blue); (3) explore predictors of drivers’ trust in ADAS through a mixed linear model (highlighted in green). All analyses were conducted in SAS OnDemand for Academics.

As a first step, a cluster analysis using the PROC CLUSTER procedure was conducted based on OMMS and four gbMMS scores. Specifically, Ward’s Hierarchical Clustering method was adopted (Johnson and Wichern, 1992), with the distances between clusters computed using Ward’s minimum-variance method, and the data standardized with the STD option in PROC CLUSTER. The number of clusters was chosen based on the metric of semi-partial R-squared (Kaufman and Rousseeuw, 2009).

For Step 2, to explore the predictors of drivers’ ADAS mental model, we fitted a logistic regression model with PROC LOGISTIC. The clusters identified from the previous step were used as the dependent variable. The demographic variables (e.g., age), driving habits-related variables (e.g., driving experience), driving styles, personalities, and their two-way interactions were used as the independent variables in the initial full model. Then a backward stepwise selection method based on Bayesian Information Criterion (BIC) (Burnham and Anderson, 2004) was used for model selection.

Finally, for Step 3, to identify the predictors of drivers’ trust in ADAS, a mixed linear model was fitted using PROC MIXED with the trust in ADAS as the dependent variable. The cluster (i.e., ADAS mental model group) was included in the model as an independent variable. We used cluster instead of raw mental model scores as the five mental model scores (OMMS score and 4 gbMMS scores) are highly correlated (e.g., OMMS and gbMMS-ACC: r (285) = 0.74, *p* < 0.0001; gbMMS-ACC and gbMMS-LCC: r (285) = 0.29, *p* < 0.0001; gbMMS-ACC and gbMMS-limitation: r (285) = 0.35, *p* < 0.0001) – including all of them in the model would cause collinearity issue; while information might be lost if we include only one of the scores in the model. Further, to avoid potential collinearity issues, all variables that were significant in the logistic regression model in Step 2 were excluded as independent variables in Step 3. The two-way interactions of the independent variables were also included in the initial full model. Similarly, we applied the backward stepwise selection method for model selection.

## 4. Results

### 4.1. Descriptions of ADAS mental model

**Figure 2:**
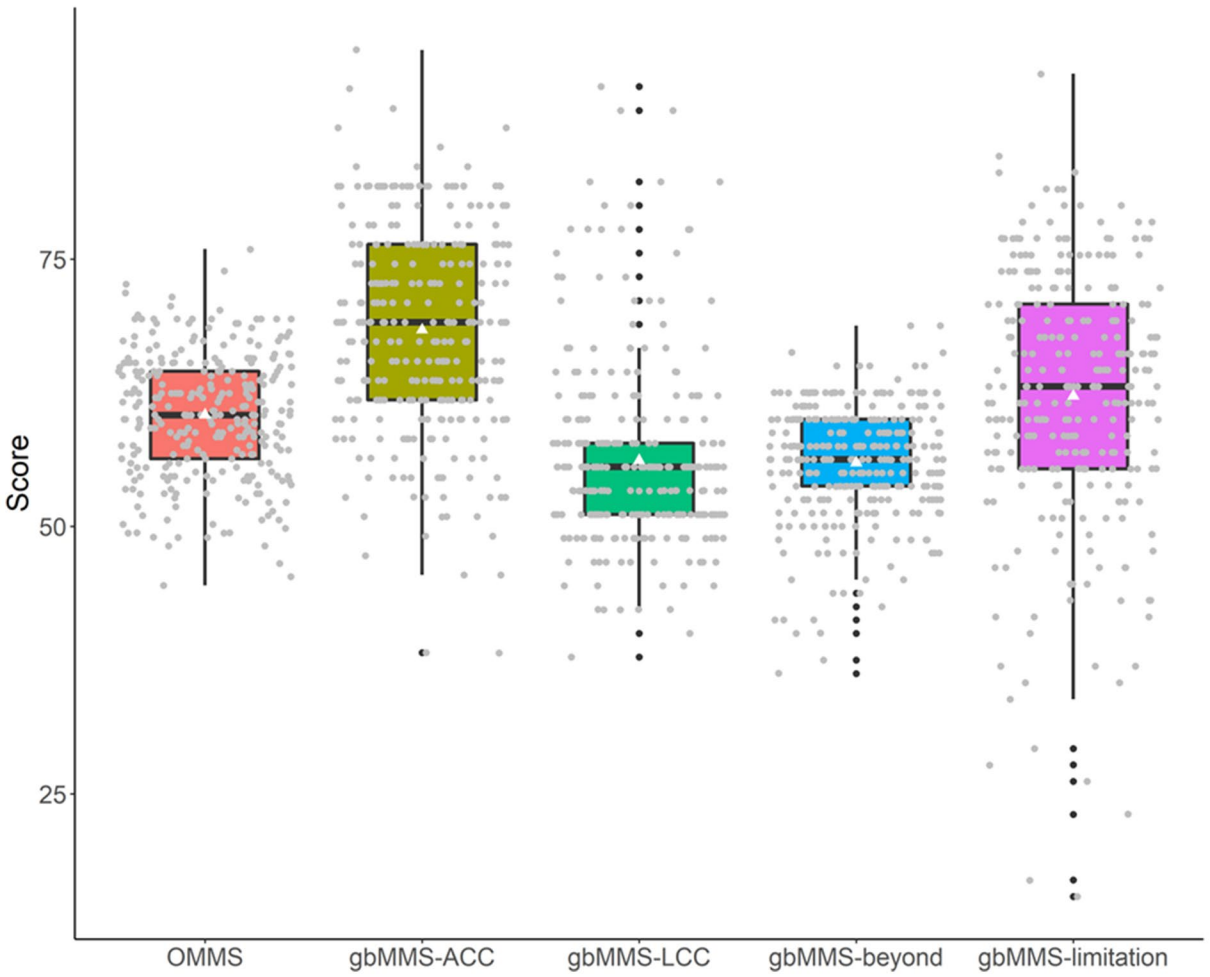
Mental model scores of different groups of questions. In this and the following plots, box plots present the minimum, 1st quartile, median, 3rd quartile, and maximum, along with the mean depicted through a white triangle. The mean (M) and standard deviation (SD) are provided at the top of each plot. Nonsignificant pairwise comparisons are marked as “ns” in red.

The mental model scores are summarized in Table 1 and visualized in Figure 2. Through ANOVA with repeated measures, a significant difference has been observed between mental model scores (*F*(4,1,430) = 100.74, *p* < 0.0001). As shown in Table 2, pairwise comparisons were made between mental model scores. Drivers in general had a better knowledge of ACC functions compared to that of LCC functions and functions beyond ACC & LCC. Further, drivers’ knowledge of ADAS limitations is at a medium level but a large variation has been observed in samples, indicating the existence of other underlying influential predictors of driver’ knowledge of the ADAS limitations.

**Table 2:** Pairwise comparisons of mental model scores between groups.

Pairwise comparisons	Compared to			
	OMMS	gbMMS-ACC	gbMMS-LCC	gbMMS-Beyond
gbMMS-ACC	*t*(1430) = 11.0 *p* < 0.0001 ∆ = 7.9 (95%CI: [6.5, 9.3])	**/**	**/**	**/**
gbMMS-LCC	*t*(1430) = −5.9 *p* < 0.0001 ∆ = −4.3 (95%CI: −5.7, −2.9)	*t*(1430) = −17.0 *p* < 0.0001 ∆ = −12.2 (95%CI: [−13.6, −10.8])	/	/
gbMMS-Beyond	*t*(1430) = −6.2 *p* < 0.0001 ∆ = −4.5 (95%CI: [−5.9, −3.1])	*t*(1430) = −17.3 *p* < 0.0001 ∆ = −12.4 (95%CI: [−13.8, −11.0])	N.S.	/
gbMMS-limitation	*t*(1430) = 2.4 *p* = 0.02 ∆ = 1.8 (95%CI: [0.4, 3.2])	*t*(1430) = −8.6 *p* < 0.0001 ∆ = −6.2 (95%CI: [−7.6, −4.8])	*t*(1430) = 8.4 *p* < 0.0001 ∆ = 6.0 (95%CI: [4.6, 7.4])	*t*(1430) = 8.7 *p* < 0.0001 ∆ = 6.2 (95%CI: [4.8, 7.6])

### 4.2. Cluster analysis

**Figure 3:**
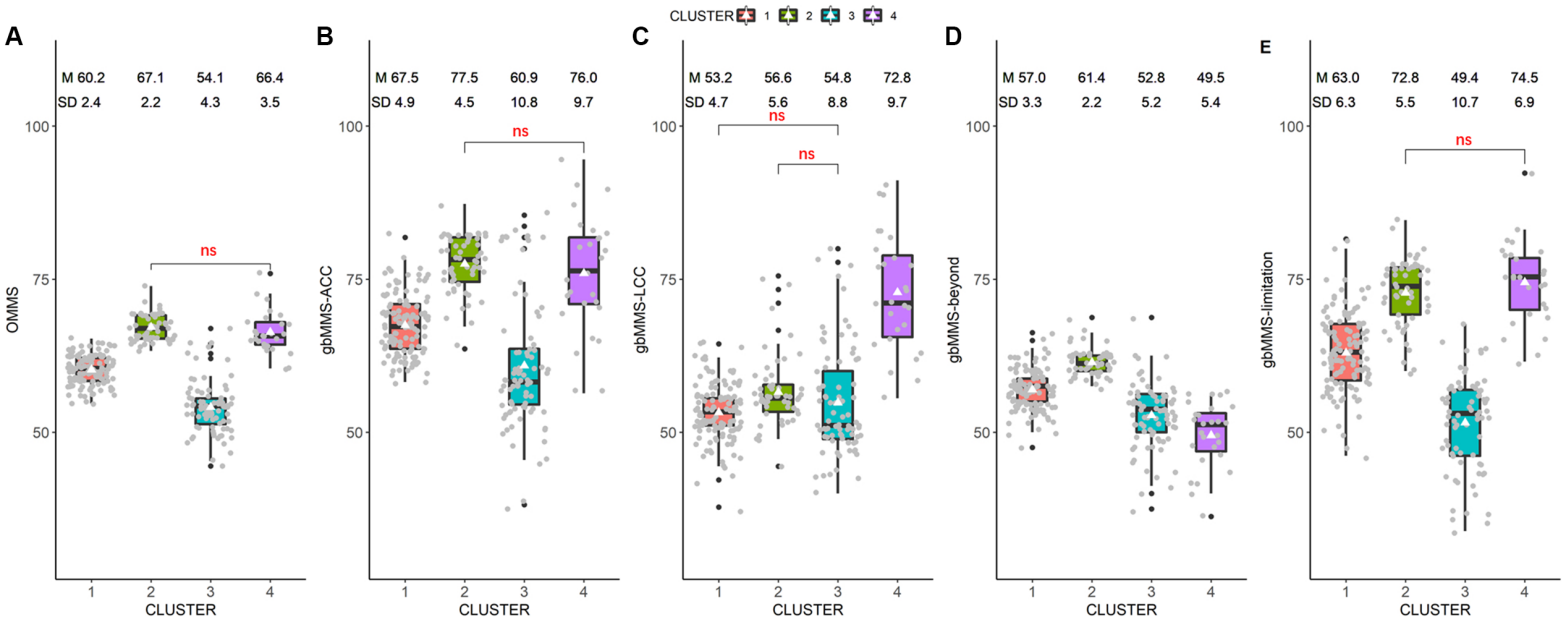
Comparisons between different clusters: **(A)** OMMS; **(B)** gbMMS-ACC; **(C)** gbMMS-LCC; **(D)** gbMMS-beyond; **(E)** gbMMS-limitation.

Through cluster analysis, 4 clusters with different ADAS mental model characteristics were identified (cluster 1: *N* = 124, cluster 2: *N* = 56, cluster 3: *N* = 80, cluster 4: *N* = 27). Figure 3 presents the mental scores within each group and Table 3 presents the pairwise comparisons of the mental scores between clusters.

**Table 3:** Pairwise comparisons of mental model scores between clusters.

Statistics		Compared to		
		Cluster 1	Cluster 2	Cluster 3
Pairwise comparisons for **OMMS**	Cluster**2**	*t*(283) = 13.8, *p* < 0.0001 ∆ = 6.9 (95%CI: [5.9, 7.9])	**/**	**/**
	Cluster**3**	*t*(283) = −13.8, *p* < 0.0001 ∆ = −6.1 (95%CI: −7.0, −5.2)	*t*(283) = −24.1, *p* < 0.0001 ∆ = −13.1 (95%CI: [−14.2, −12.0])	/
	Cluster**4**	*t*(283) = 9.3, *p* < 0.0001 ∆ = 6.1 (95%CI: [4.8, 7.4])	N.S.	*t*(283) = 17.8, *p* < 0.0001 ∆ = 12.3 (95%CI: [10.9, 13.7])
Pairwise comparisons for **gbMMS-ACC**	Cluster**2**	*t*(283) = 8.4, *p* < 0.0001 ∆ = 10.1 (95%CI: [7.7, 12.5])	**/**	**/**
	Cluster**3**	*t*(283) = −6.2, *p* < 0.0001 ∆ = −6.6 (95%CI: [−8.7, −4.5])	*t*(283) = −12.8, *p* < 0.0001 ∆ = −16.6 (95%CI: [−19.1, −14.1])	/
	Cluster**4**	*t*(283) = 5.4, *p* < 0.0001 ∆ = 8.5 (95%CI: [5.4, 11.6])	N.S.	*t*(283) = 9.1, *p* < 0.0001 ∆ = 15.1 (95%CI: [11.8, 18.4])
Pairwise comparisons for **gbMMS-LCC**	Cluster**2**	*t*(283) = 3.1, *p* = 0.003 ∆ = 3.3 (95%CI: [1.1, 5.5])	/	/
	Cluster**3**	N.S.	N.S.	/
	Cluster**4**	*t*(283) = 13.6, *p* < 0.0001 ∆ = 19.6 (95%CI: [16.8, 22.4])	*t*(283) = 10.2, *p* < 0.0001 ∆ = 16.3 (95%CI: [13.2, 19.4])	*t*(283) = 11.9, *p* < 0.0001 ∆ = 18.0 (95%CI: [15.0, 21.0])
Pairwise comparisons for **gbMMS-beyond**	Cluster**2**	*t*(283) = 7.0, *p* < 0.0001 ∆ = 4.5 (95%CI: [3.2, 5.8])	/	/
	Cluster**3**	*t*(283) = −7.3, *p* < 0.0001 ∆ = −4.2 (95%CI: [−5.3, −3.1])	*t*(283) = −12.5, *p* < 0.0001 ∆ = −8.7 (95%CI: [−10.1, −7.3])	/
	Cluster**4**	*t*(283) = −8.8, *p* < 0.0001 ∆ = −7.5 (95%CI: [−9.2, −5.8])	*t*(283) = −12.8, *p* < 0.0001 ∆ = −11.9 (95%CI: [−13.8, −10.0])	*t*(*t*(283) = −3.7, *p* = 0.0003 ∆ = −3.3 (95%CI: [−5.0, −1.6])
Pairwise comparisons for **gbMMS-limitation**	Cluster**2**	*t*(283) = 7.9, *p* < 0.0001 ∆ = 9.8 (95%CI: [7.4, 12.2])	/	/
	Cluster**3**	*t*(283) = −12.2, *p* < 0.0001 ∆ = −13.5 (95%CI: [−15.7, −11.3])	*t*(283) = −17.4, *p* < 0.0001 ∆ = −23.3 (95%CI: [−26.0, −20.6])	/
	Cluster**4**	*t*(283) = 7.1, *p* < 0.0001 ∆ = 11.5 (95%CI: [8.3, 14.7])	N.S.	*t*(283) = 14.6, *p* < 0.0001 ∆ = 25.0 (95%CI: [21.6, 28.4])

**Figure 4:**
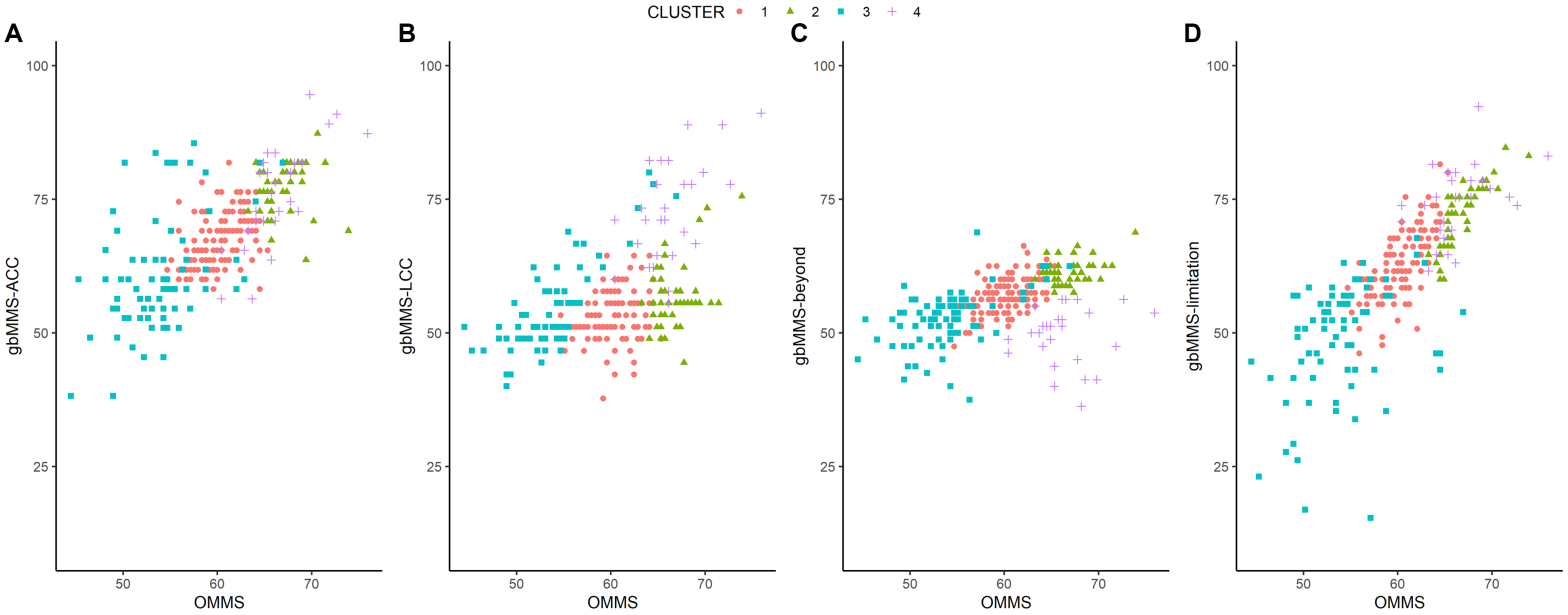
The relationship between OMMS and four gbMMSs in four clusters: **(A)** gbMMS-ACC; **(B)** gbMMS-LCC; **(C)** gbMMS-beyond; **(D)** gbMMS-limitation.

To further illustrate the characteristics of the clusters, the gbMMS scores were plotted against the OMMS score in Figure 4. Based on the distributions of the mental scores, we name the four clusters as follows:

Weak mental model (WMM) group (i.e., Cluster 3): Drivers in this group has a weak mental model of ADAS in terms of ADAS-related knowledge in general.

Medium mental model (MMM) group (i.e., Cluster 1): Drivers in this group has a medium level of ADAS-related knowledge in general.

Strong overall mental model but weak LCC (SMM-W-LCC) group (i.e., Cluster 2): Drivers in this group have a strong mental model of ADAS in general (OMMS). However, although the gbMMS-LCC of this cluster is the second highest among four clusters, the absolute gbMMS-LCC score is much lower than that in cluster 4. Further, this group of drivers also knew well of ADAS limitations. In summary, drivers in this group have a good understanding of ACC and ADAS limitations but a weak mental model of LCC.

Strong mental model of traditional Level-2 ADAS (SMM-ACC/LCC) group (i.e., Cluster 4): Drivers in this group have a strong mental model of ADAS in general (OMMS) and know traditional Level-2 ADAS (ACC and LCC) well, though they have relatively weak knowledge of functions beyond ACC & LCC (gbMMS-Beyond). However, it should be noted that all clusters have relatively low gbMMS-Beyond scores and the differences between clusters are small. Thus, this group (Cluster 4) can still be regarded as having the strongest mental model of advanced Level-2 ADAS.

### 4.3. Factors associated with drivers’ mental model of ADAS

Table 4 presents the Wald statistics of type 3 analysis for the multinomial logistic regression model. It is found that the years of licensure, average driving distance, ADAS familiarity, planning, and agreeableness are significant predictors of clusters.

**Table 4:** Wald statistics of type 3 analysis for the logistic regression model.

IV	*χ*^2^-value	*p*
Years of licensure	*χ*^2^(3) = 9.87	0.02^*^
Weekly driving distance	*χ*^2^(6) = 13.98	0.03^*^
Technology familiarity	*χ*^2^(3) = 7.45	0.06
ADAS familiarity	*χ*^2^(3) = 35.27	<0.0001^*^
Planning	*χ*^2^(3) = 9.72	0.02^*^
Agreeableness	*χ*^2^(3) = 10.39	0.02^*^

In this table and the following tables, *marks significant effects. IV stands for the independent variable.

Post-hoc comparisons show that, for every 1-year increase in the Years of licensure, the odds ratio (OR) of belonging to the MMM group versus the WMM group was 0.90, with a 95% confidence interval (CI) of [0.82, 0.99]. In other words, drivers with longer years of licensure tended to have a weaker mental model.

Further, compared to drivers who drove over 300 km per week, drivers who drove less than 99 km per week had a lower likelihood of belonging to the SMM-W-LCC group and SMM-ACC/LCC versus WMM, with odds ratios (ORs) of 0.21 (95%CI: [0.04, 0.98]) and 0.016 (95%CI: [0.001, 0.187]), respectively. This indicates that drivers who drove more had better ADAS mental models compared to those who drove less in general.

As for the effects of familiarity, with every 1-unit increase in the ADAS familiarity, drivers were more likely to belong to the SMM-W-LCC group compared to belonging to the WMM group (OR = 5.50, 95%CI: [2.79, 10.82]) and MMM group (OR = 4.50, 95%CI: [2.53, 8.03]). Therefore, drivers’ mental models of ADAS would generally improve with the increase of self-reported ADAS familiarity.

As for the influence of driving style, every 1-unit increase in Planning (a division of driving style) led to a 1.54 (95%CI: [1.04, 2.26]) multiplicative increase in the odds of belonging to the SMM-ACC/LCC group versus to WMM group. At the same time, every 1-unit increase of the Planning led to a higher likelihood of belonging to SMM-W-LCC (OR = 1.36, 95%CI: [1.05, 1.76]) and SMM-ACC/LCC (OR = 1.57, 95%CI: [1.09, 2.25]) versus belonging to MMM group. In general, it seems that drivers who preferred planning in advance during a trip tended to have a stronger mental model of ADAS.

Regarding the personality traits, with every 1-unit increase in Agreeableness, drivers were less likely to belong to SMM-ACC/LCC group versus the MMM group (OR = 0.30, 95%CI: [0.14, 0.64]) and WMM group (OR = 0.38, 95%CI: [0.17, 0.84]). Therefore, drivers with higher agreeableness would generally have a weaker mental model of ADAS.

### 4.4. Factors associated with drivers’ trust in ADAS

**Figure 5:**
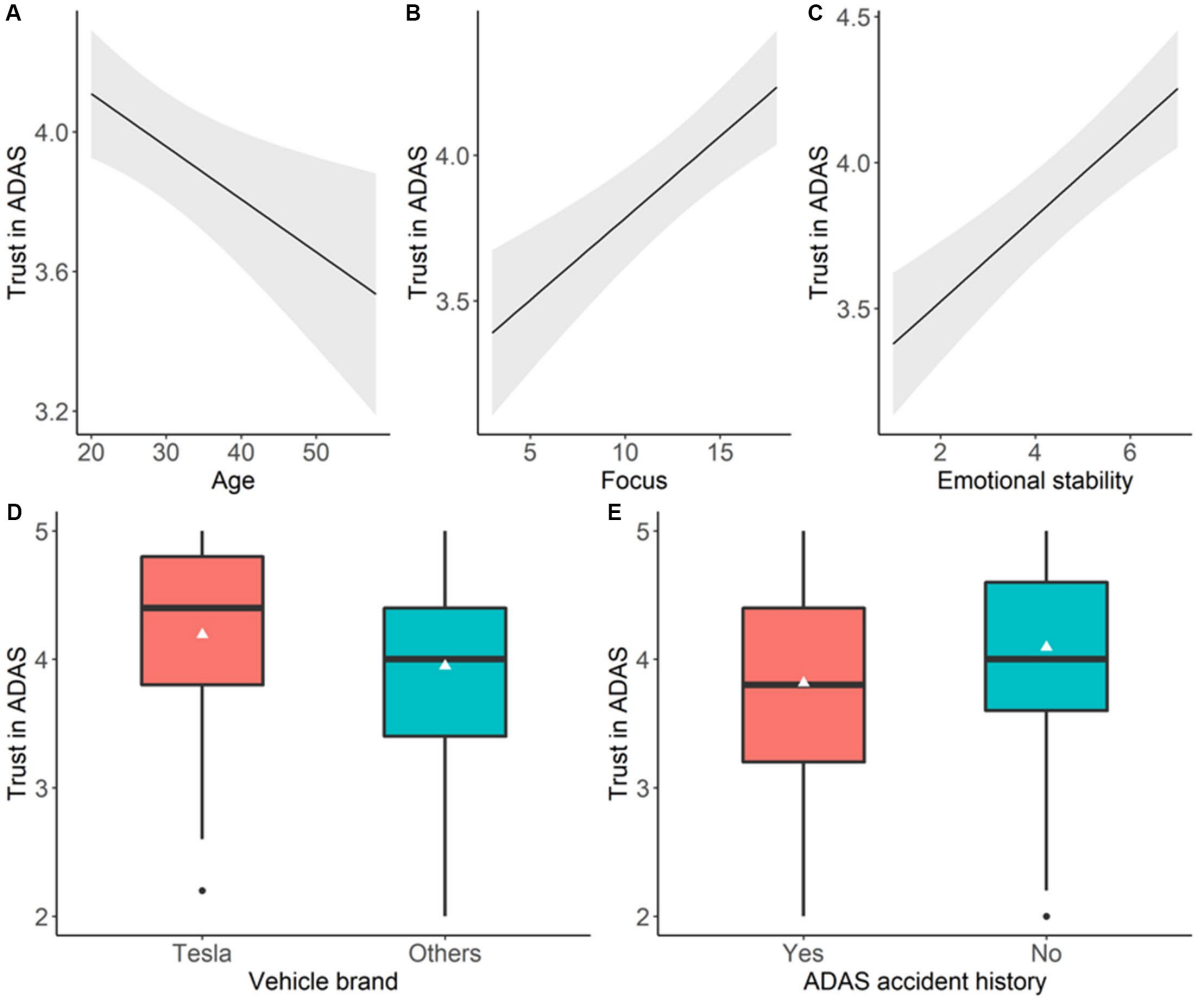
Illustration of drivers’ trust in ADAS representing significant main effects. In **(A-C)**, the shaded area represents 95% confidence intervals. **(A)** Age; **(B)** Focus; **(C)** Emotional stability; **(D)** Vehicle brand; **(E)** ADAS accident history.

Table 5 presents the Type 3 tests of fixed effects for the mixed model. It was found that the cluster (i.e., mental model group), vehicle brand, age, education, ADAS experience, ADAS accident history, focus, and emotional stability were significant predictors of drivers’ trust in ADAS. Figure 5 illustrates the main effects of significant predictors on drivers’ trust in ADAS.

**Table 5:** Type 3 tests of fixed effects for the mixed linear model.

IV	*F*-value	*p*
Group	*F* (3, 266) = 9.94	<0.0001^*^
Vehicle brand	*F* (1, 266) = 9.62	0.002^*^
Age	F (1, 266) = 7.66	0.006^*^
ADAS experience	F (3, 266) = 4.91	0.003^*^
Group*ADAS experience	*F* (9, 266) = 2.52	0.009^*^
ADAS accident history	F (1, 266) = 5.52	0.02^*^
Focus	F (1, 266) = 21.35	<0.0001^*^
Emotional stability	F (1, 266) = 29.02	<0.0001^*^

Specifically, as shown in Figure 5, it was found that age was negatively correlated with drivers’ trust in ADAS: with every 1-year increase in age, drivers’ trust in ADAS decreased 0.015 units (t(266) = −2.77, *p* = 0.006, 95%CI: [0.004, 0.026]). Further, with every 1-unit increase in the *Focus* division of driving style, drivers would have 0.056 units (t(266) = 4.62, *p* < 0.0001, 95%CI: [0.032, 0.080]) increase in trust in ADAS. At the same time, every 1-unit increase in emotional stability led to 0.146 units (t(266) = 5.39, *p* < 0.0001, 95%CI: [0.093, 0.199]) increase in drivers’ trust in ADAS. At the same time, it was found that drivers who encountered ADAS-related accidents trusted less in ADAS compared to drivers without ADAS-related accident history (∆ = −0.26, 95%CI: [−0.48, −0.04], t (266) = −2.35, *p* = 0.02). Besides, Tesla owners showed significantly higher trust in ADAS compared to owners of vehicles with other brands (∆ = 0.21, 95%CI: [0.08, 0.34], *t* (266) = 3.10, *p* = 0.002).

**Figure 6:**
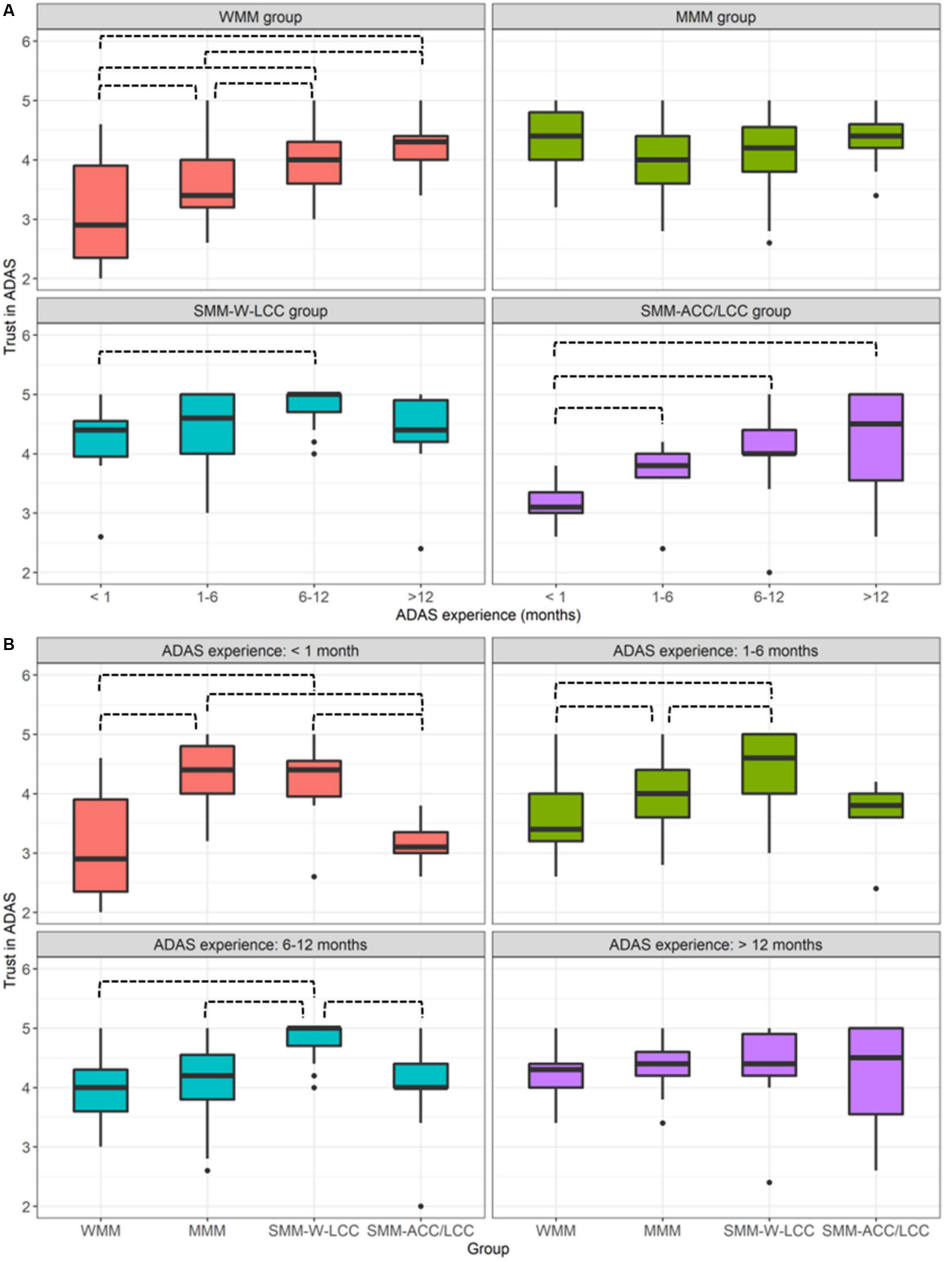
Illustration of drivers’ trust in ADAS representing the significant interaction effect between mental model group and ADAS experience: **(A)** grouped by mental model groups; **(B)** grouped by ADAS experience. All significant pairwise comparisons are marked using black dashed lines.

The interaction effect between *Group* and *ADAS experience* is illustrated in Figure 6, and the corresponding pairwise comparisons are summarized in Tables 6, 7. With more ADAS experience, drivers in the WMM group and SMM-ACC/LCC group tended to have higher trust in ADAS (Figure 6A). When comparisons were made across mental model groups, it seems that with increased ADAS experience, the association between mental model group and trust became weaker. Specifically, with less than one month of ADAS experience, the middle level of the mental model was associated with a high trust in ADAS, while no significant pairwise comparisons were observed with over 12 months of ADAS experience.

**Table 6:** Pairwise comparisons of trust scores between ADAS experience levels given mental model groups.

Mental model of ADAS	ADAS experience	Compared to		
		< 1 month	1–6 months	6–12 months
WMM	1–6 months	*t*(266) = 2.0, *p* = 0.04 ∆ = 0.5 (95%CI: [0.1, 0.9])	/	/
	6–12 months	*t*(266) = 3.1, *p* = 0.002 ∆ = 0.8 (95%CI: [0.3, 1.3])	*t*(266) = 2.1, *p* = 0.04 ∆ = 0.3 (95%CI: [0.1, 0.5])	/
	>12 months	*t*(266) = 3.2, *p* = 0.001 ∆ = 0.9 (95%CI: [0.4, 1.4])	*t*(266) = 2.2, *p* = 0.03 ∆ = 0.4 (95%CI: [0.1, 0.7])	N.S.
MMM	1–6 months	N.S.	/	/
	6–12 months	N.S.	N.S.	/
	>12 months	N.S.	N.S.	N.S.
SMM-W-LCC	1–6 months	N.S.	/	/
	6–12 months	*t*(266) = 2.0, *p* = 0.04 ∆ = 0.5 (95%CI: [0.1, 0.9])	N.S.	/
	>12 months	N.S.	N.S.	N.S.
SMM-ACC/LCC	1–6 months	*t*(266) = 2.8, *p* = 0.005 ∆ = 0.9 (95%CI: [0.3, 1.5])	/	/
	6–12 months	*t*(266) = 3.3, *p* = 0.001 ∆ = 1.0 (95%CI: [0.4, 1.6])	N.S.	/
	>12 months	*t*(266) = 3.5, *p* = 0.0006 ∆ = 1.1 (95%CI: [0.5, 1.7])	N.S.	N.S.

**Table 7:** Pairwise comparisons of trust scores between mental model groups given ADAS experience levels.

ADAS experience	Mental model groups	Compared to		
		WMM	MMM	SMM-W-LCC
<1 month	MMM	*t*(266) = 3.7, *p* = 0.0003 ∆ = 1.2 (95%CI: [0.6, 1.8])	**/**	**/**
	SMM-W-LCC	*t*(266) = 2.5, *p* = 0.02 ∆ = 0.8 (95%CI: [0.2, 1.4])	N.S.	/
	SMM-ACC/LCC	N.S.	*t*(266) = −4.2, *p* < 0.0001 ∆ = −1.4 (95%CI: [−2.1, −0.7])	*t*(266) = −3.0, *p* = 0.003 ∆ = −0.9 (95%CI: [−1.5, −0.3])
1–6 months	MMM	*t*(266) = 2.6, *p* = 0.01 ∆ = 0.3 (95%CI: [0.1, 0.5])	**/**	**/**
	SMM-W-LCC	*t*(266) = 4.4, *p* < 0.0001 ∆ = 0.7 (95%CI: [0.4, 1.0])	*t*(266) = 2.6, *p* = 0.01 ∆ = 0.4 (95%CI: [0.1, 0.7])	/
	SMM-ACC/LCC	N.S.	N.S.	N.S.
6–12 months	MMM	N.S.	/	/
	SMM-W-LCC	*t*(266) = 3.0, *p* = 0.003 ∆ = 0.5 (95%CI: [0.2, 0.8])	*t*(266) = 2.9, *p* = 0.004 ∆ = 0.5 (95%CI: [0.2, 0.8])	/
	SMM-ACC/LCC	N.S.	N.S.	*t*(266) = −2.2, *p* = 0.03 ∆ = −0.5 (95%CI: [−0.9, −0.1])
>12 months	MMM	N.S.	/	/
	SMM-W-LCC	N.S.	N.S.	/
	SMM-ACC/LCC	N.S.	N.S.	N.S.

## 5. Discussion

Through a survey study among 287 ADAS users, we assessed drivers’ mental models of advanced ADAS and further investigated how mental models along with other underlying factors may affect drivers’ trust in ADAS. Being different from previous research that mainly focused on traditional ADAS technologies, our study targeted toward advanced Level-2 ADAS (e.g., Autopilot in Tesla) and paid additional attention to ADAS functions beyond ACC and LCC (e.g., automatic lane changing), considering the expanding market share of them. This study provides evidence to support the role of culture difference on influencing users’ trust in ADAS, by analyzing the survey data collected from the users of one of the largest ADAS markets in the world.

### 5.1. Drivers’ mental model of ADAS

In general, we notice that drivers had better knowledge of ACC compared to that of LCC and the functions beyond ACC and LCC. The relatively weak mental model of the functions beyond ACC and LCC is in line with our expectations. First, drivers might be less familiar with emerging functions that are only available in the past few years. Second, the capabilities of these emerging functions vary across vehicle brands and even change with time–vehicle manufacturers update vehicle functions Over the Air (OTA), increasing the learning cost of consumers. However, the weak mental model of LCC is beyond our expectations. We notice that previous research focusing on drivers’ ADAS mental models did not reveal this significant difference between ACC and LCC (DeGuzman and Donmez, 2021a). A potential explanation is that our study targeted toward a different population compared to those of previous studies. The discrepancies in vehicle culture, traffic rules, advertising strategies, and market shares of these systems may contribute to the difference in mental models. However, it should be noted that it would be unfair to compare the mental model in our study with the mental model in previous studies directly, as different scoring strategies were adopted. A future study targeted toward driver populations in different countries may further reveal whether population differences exist and what the underlying reasons leading to these differences, which can guide the design of customized driver training programs and advertising strategies in different markets.

Further, through cluster analysis, we identified four driver groups in terms of their mental models of ADAS. In general, no group (nor drivers) reached a literally high score for functions beyond ACC and LCC (the maximum obtained score is below 70 out of 100). Specifically, 80 out of 287 (28%) drivers were classified as having a weak mental model (WMM group) with low mental scores of all types of ADAS functions and limitations; 56 (20%) drivers in the SMM-L-LCC group had good knowledge of ACC but weak knowledge of LCC. Only 27 (9%) drivers in SMM-ACC/LCC group had good knowledge of ACC and LCC (i.e., functions of traditional Level-2 ADAS).

At the same time, several factors have been found to be associated with driver grouping in terms of the mental model. First, as expected, drivers with higher self-reported ADAS familiarity are more likely to have strong mental models of ADAS, indicating a consistency between subjective evaluation (i.e., self-reported ADAS familiarity) and objective assessment (i.e., mental model scores). Thus, it seems to be practically feasible to roughly evaluate drivers’ mental models through a single question. However, further validation through more carefully designed studies is needed before we can draw a conclusion.

Further, drivers with longer years of licensure were found to be more likely to belong to the WMM group compared to the MMM group – or in other words, to have a weaker ADAS mental model. The years of licensure have been found to be correlated with the age of drivers inherently (McCartt et al., 2003; Curry et al., 2015), which is also the case in our dataset (correlation between age and years of licensure: *r* (285) = 0.58, *p* < 0.0001). Thus, the relationship between years of licensure and mental model performance might be the effect of age – those who are younger tend to be earlier adopters of new technologies (Lu et al., 2009) and thus may have better knowledge of the ADAS system. However, the readers should be cautious about this interpretation as other potential covariates might also influence the relationship we have observed. For example, it is possible that the driver training program has been changing in the past years, which may lead to a difference in drivers’ understanding of the ADAS. Future research is needed to investigate these relationships with better control of the covariates. Besides, it was found that drivers who drove more mileage (over 300 km/week) tended to have stronger mental models of ADAS compared to those who drive less mileage (less than 99 km/week). It is possible that those who drove more mileage (over 300 km/week) tended to use ADAS for long trips and thus they could gain more experience with ADAS. It should be noted that, as we distributed the survey online, there might be professions (e.g., truck drivers) in our respondents. These drivers may drive more but have constructed stronger ADAS mental models through professional training, which may bias our interpretation between driving experience and mental model. Future research with better controlled respondent population or larger sample size is needed to further validate the relationship we have observed.

In terms of driving style and personality, drivers who had higher planning scores (i.e., tend to plan the trip for an unfamiliar long journey) and with lower agreeableness scores (i.e., tend to be more competitive and sometimes even manipulative) were more likely to have strong mental models of ADAS. Previous research found that agreeableness is positively correlated with the adoption of 5G technology (Irfan and Ahmad, 2022). It is possible that those who had higher planning scores and who were more open to technology would prefer to actively seek information regarding ADAS. This finding highlights the importance of providing customized ADAS training to different user groups in order to calibrate their mental model of ADAS.

### 5.2. Drivers’ trust in ADAS

First, it is interesting to notice that drivers’ mental model was associated with users’ trust in ADAS when the users had little experience with ADAS, while the association became weaker after one year of ADAS usage. More specifically, with increased ADAS experience, for drivers with weak or strong mental models, their trust in ADAS showed a generally increasing trend, and drivers in all mental model groups reached a similar level of trust in ADAS after one year of usage. This result echoes the findings in DeGuzman and Donmez (2021b) but our findings provide a higher resolution. It seems that when there is little or no experience (i.e., non-owners or new users), drivers’ trust in ADAS is dependent on their knowledge of ADAS; and the trust would gradually increase and become stable after they gained more experience with the ADAS – this trend of trust is decoupled with the variations in the mental model. The increase in trust with the increase of experience with the system has been widely observed in previous studies (e.g., Beggiato et al., 2015). Our results only partially support this conclusion (for drivers who had very strong mental models or very weak mental models) but further indicate that the increase of trust with the accumulation of experience may not be the result of increased knowledge in the system. Further studies are needed to explore other factors that can explain the trend of trust in automation with the accumulation of experience.

Further, being different from what has been found in DeGuzman and Donmez (2021a), our findings reveal that the relationship between the mental model and initial trust in ADAS (less than 1 month of ADAS experience) is not monotonic. Drivers with a middle level of ADAS knowledge reported the highest trust in ADAS. This might because that in our dataset, drivers with weak mental models are in general with longer years of licensure and are relatively older. Our study, along with some previous research found that age is negatively correlated with trust in technology (Dikmen and Burns, 2017), which may explain the relatively low level of trust in ADAS among drivers with weak ADAS mental model. As for drivers with strong mental models, they might be more aware of the system’s limitations compared to those with middle-level mental models and thus they might trust less in ADAS.

At the same time, as expected, we found that ADAS-related accidents led to lower trust in ADAS, which is in line with previous studies (Yu et al., 2017; Lee et al., 2021). It was also found that drivers with higher focus (i.e., higher resistance to driving distraction) and with higher emotional stability (i.e., lower predisposition to psychological stress) would have higher trust in ADAS. The consistency of the driving styles between driving automation and drivers was found to influence drivers’ trust in the driving automation (Ma and Zhang, 2020) and the personality can be associated with drivers’ dispositional trust in automation (Hoff and Bashir, 2015), which may explain the associations among driving style, personality, and trust in ADAS observed in our study. However, future research is needed to further explore these relationships. We also noticed that drivers of different brands of the vehicle had different levels of trust in ADAS. This might be attributed to different system designs, advertising strategies, and user populations. For example, Tesla has very different in-vehicle human machine interface (HMI) designs compared to vehicles made by traditional manufacturers. Although we cannot make comparisons between the HMIs in different vehicles with the data collected in our study, the transparency (Yang et al., 2017) and usability of the HMIs (Acemyan and Kortum, 2012) have been widely acknowledged as influential factors of trust in automation. Future research can explore underlying factors that led to the difference among users of different vehicle brands so that targeted strategies to calibrate users’ trust in ADAS can be proposed.

Interestingly, we have also observed the difference in drivers’ trust in ADAS across different countries, potentially because of the cultural difference or driver education, which deserves future investigation. Specifically, by comparing our study with other research that used the same measurement for assessing drivers’ trust in ADAS, we found that Chinese drivers generally had higher trust (Mean = 4.07, SD = 0.69) in ADAS technologies compared to drivers in U.S. (Kidd et al., 2017: Mean = 3.67, SD = 0.80) and Canada (DeGuzman and Donmez (2021a): Mean = 3.4, SD = 0.8).

Finally, it should be noted that all our analyses and discussions regarding trust are based on data from a survey study and we are not able to observe how trust may affect drivers’ behaviors when using ADAS. Thus, we are not able to assess the safety implications of our findings. Future studies may validate our findings in observational or driving simulator studies (Beggiato and Krems, 2013; Beggiato et al., 2015; Rossi et al., 2020; Zahabi et al., 2021; Pai et al., 2023). It should also be noted that all the results in this study were based on the population group of Chinese ADAS users. Future research should take potential cultural difference into consideration when interpreting or applying these findings.

## 6. Conclusion

We analyzed the survey data collected from 287 Chinese ADAS users. The survey targeted toward drivers’ mental model of and trust in emerging advanced level-2 ADAS technologies in the market. To the best of our knowledge, this is the first study targeted toward drivers’ mental model of and trust in emerging ADAS technologies beyond ACC and LCC. The findings from this study could be summarized as follows:

- By clustering drivers’ mental models of individual ADAS functions, only 27 (9%) of respondents can be categorized as having a relatively strong mental model of traditional Level-2 ADAS with ACC and LCC.
- Drivers in general had weak knowledge of LCC functions and functions beyond ACC and LCC.
- Years of licensure, weekly driving distance, ADAS familiarity, driving style (i.e., planning), and personability (i.e., agreeableness) were associated with drivers’ mental model of ADAS.
- Mental model levels, vehicle brand, and drivers’ age, ADAS experience, driving style (i.e., focus), and personality (i.e., emotional stability) were significant predictors of drivers’ trust in advanced level-2 ADAS.

The findings from this study could provide insights into the design of driver education and training programs. For example, the training programs may put more weights on LCC functions and functions beyond ACC and LCC, given that the user population has weak mental model of it in general. Further, the efforts aiming at calibrating users’ trust in ADAS may focus on more than mental model, but also need to take the characteristics of driver population into consideration, as we found that the mental model is not always associated with the variation of trust in ADAS. With more advanced technologies emerging in the field of ADAS, future research may further explore drivers’ usage behaviors of them to enhance more efficient usage of those technologies.

## Data availability statement

The raw data supporting the conclusions of this article will be made available by the authors, without undue reservation.

## Ethics statement

The studies involving human participants were reviewed and approved by Human and Artefacts Research Ethics Committee in the Hong Kong University of Science and Technology. The patients/participants provided their written informed consent to participate in this study.

## Author contributions

CH and DH: conceptualization and methodology. CH: software, formal analysis, resources, and writing—original draft preparation. CH, XW, and SY: data curation and validation. CH and XW: investigation. DH: writing—review and editing. All authors contributed to the article and approved the submitted version.

## Funding

This research was funded by the National Natural Science Foundation of China (Grant No. 52202425) and partially by the Guangzhou Municipal Science and Technology Project (No. 2023A03J0011), the Guangzhou Science and Technology Program City-University Joint Funding Project (No. 2023A03J0001), and Project of Hetao Shenzhen-Hong Kong Science and Technology Innovation Cooperation Zone (HZQB-KCZYB-2020083).

## Conflict of interest

The authors declare that the research was conducted in the absence of any commercial or financial relationships that could be construed as a potential conflict of interest.

## Publisher’s note

All claims expressed in this article are solely those of the authors and do not necessarily represent those of their affiliated organizations, or those of the publisher, the editors and the reviewers. Any product that may be evaluated in this article, or claim that may be made by its manufacturer, is not guaranteed or endorsed by the publisher.
