# Supplementary material for: Beyond adaptive cruise control and lane centering control: drivers’ mental model of and trust in emerging ADAS technologies
Source: Front Psychol. 2023 Aug 8;14:1236062. doi: 10.3389/fpsyg.2023.1236062 (PMC10442557; doi:10.3389/fpsyg.2023.1236062)
Supplement: Supplementary file 1 [file Data_Sheet_1.pdf]

Photo for Question 1

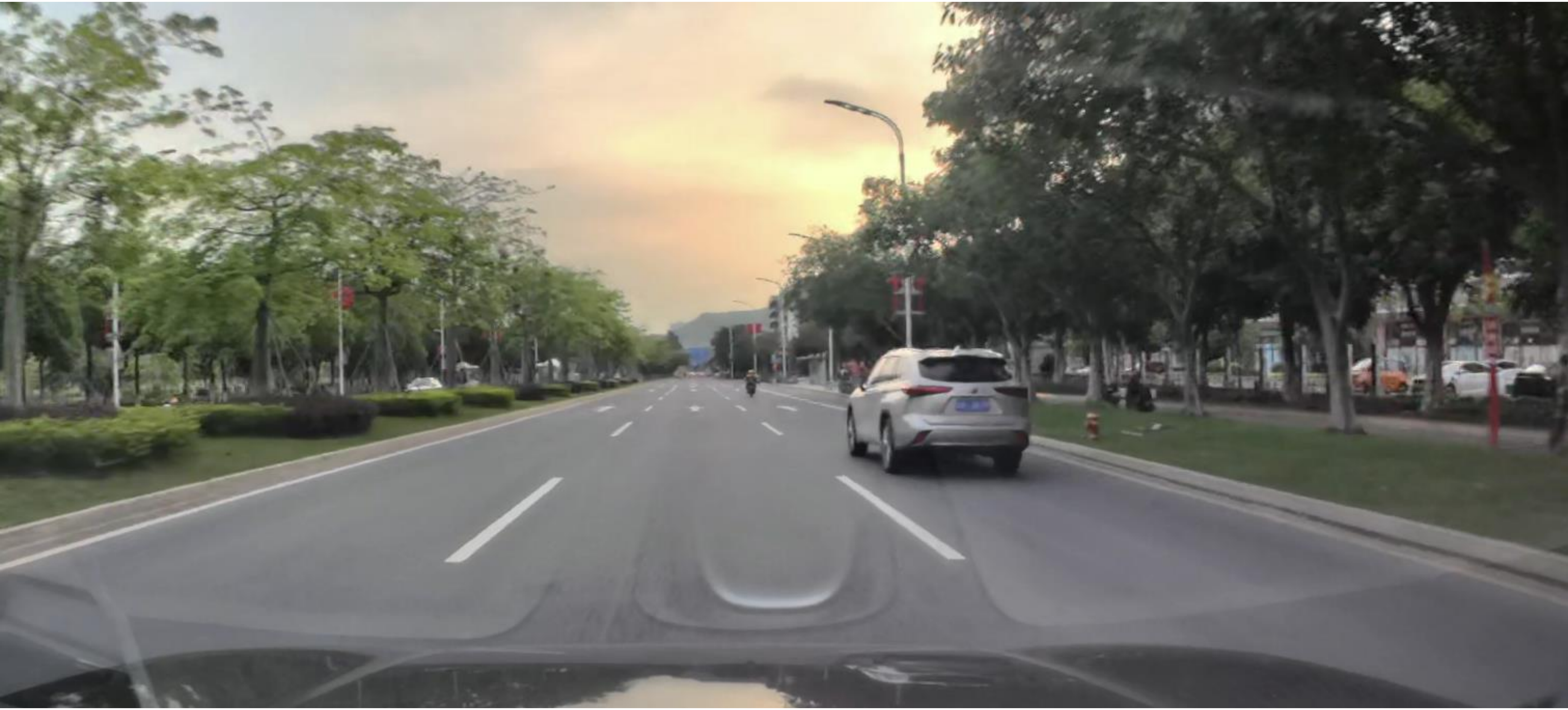

Photo for Question 2

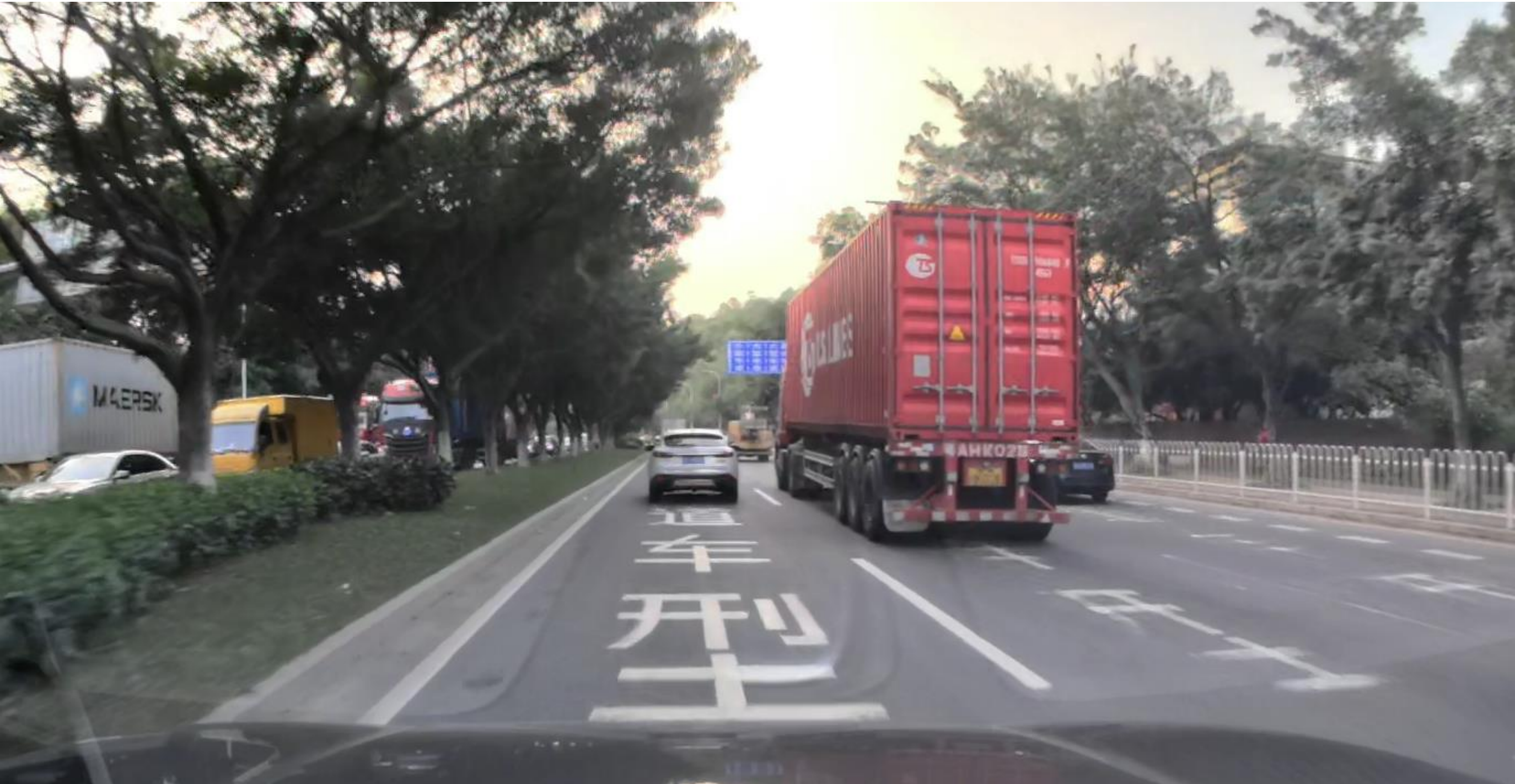

Photo for Question 4

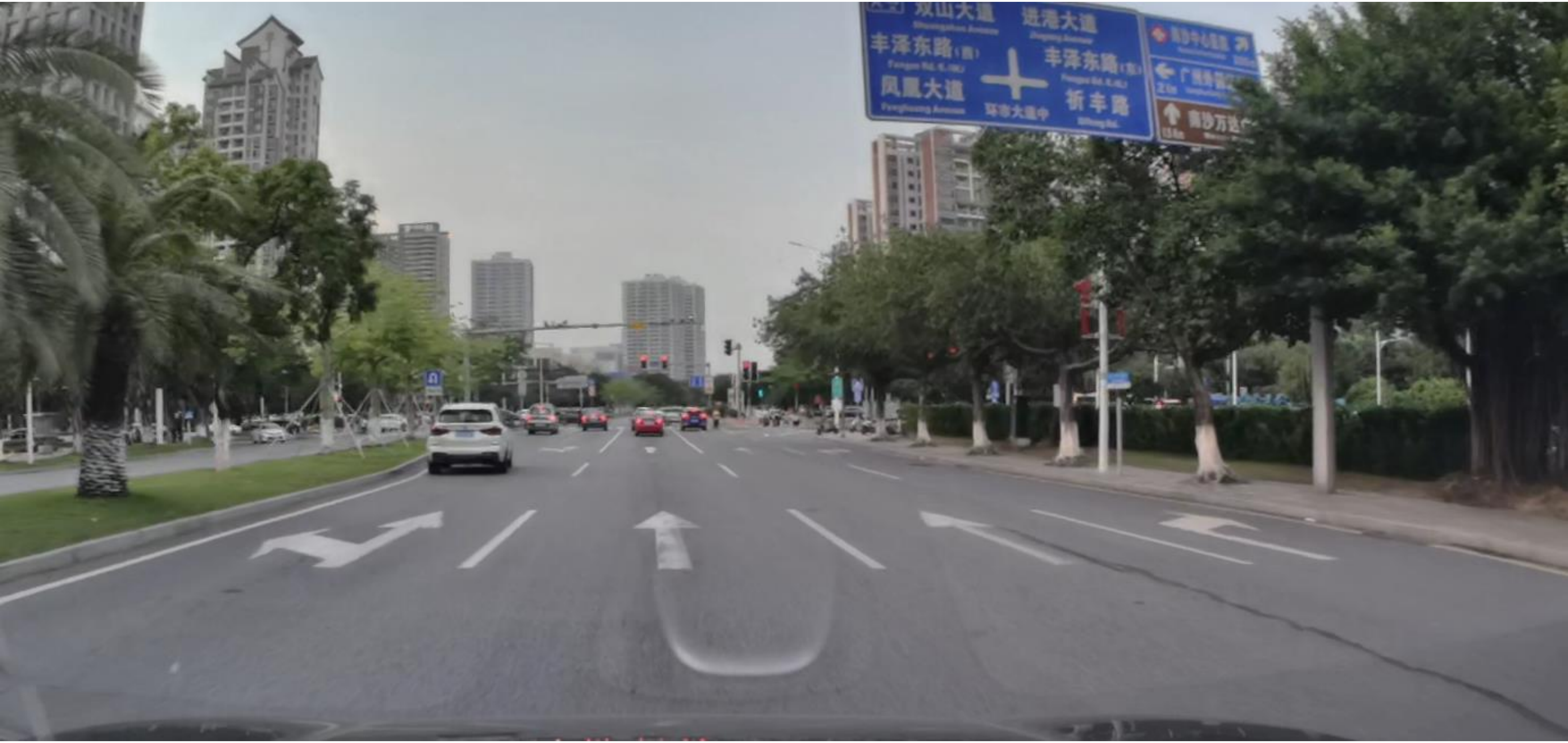

Photo for Question 5

Picture not provided for copyright issue.
If needed, please contact the author.

Photo for Question 6

Picture not provided for copyright issue.
If needed, please contact the author.

Photo for Question 7

Picture not provided for copyright issue.
If needed, please contact the author.

Photo for Question 8

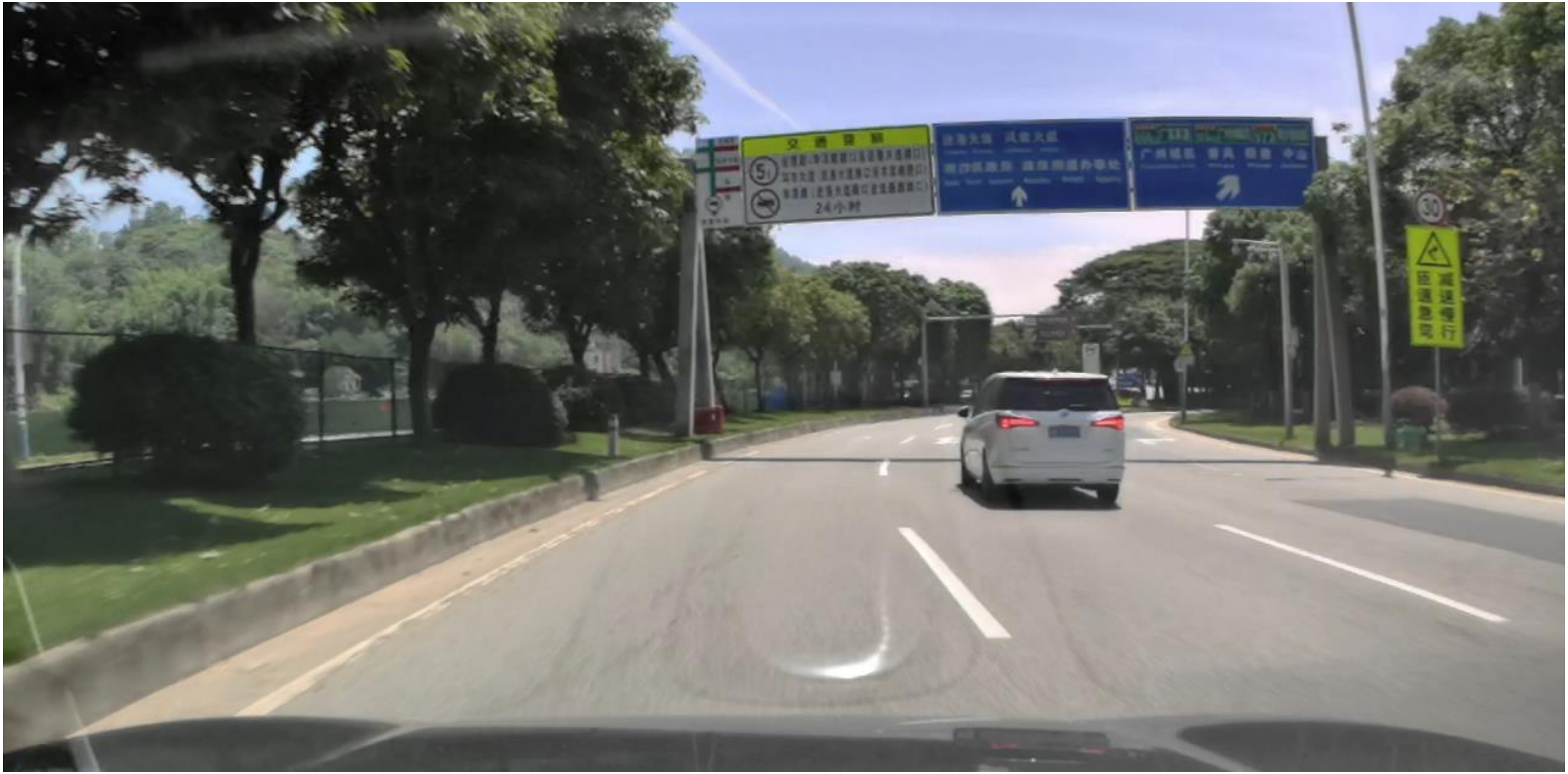

Photo for Question 10

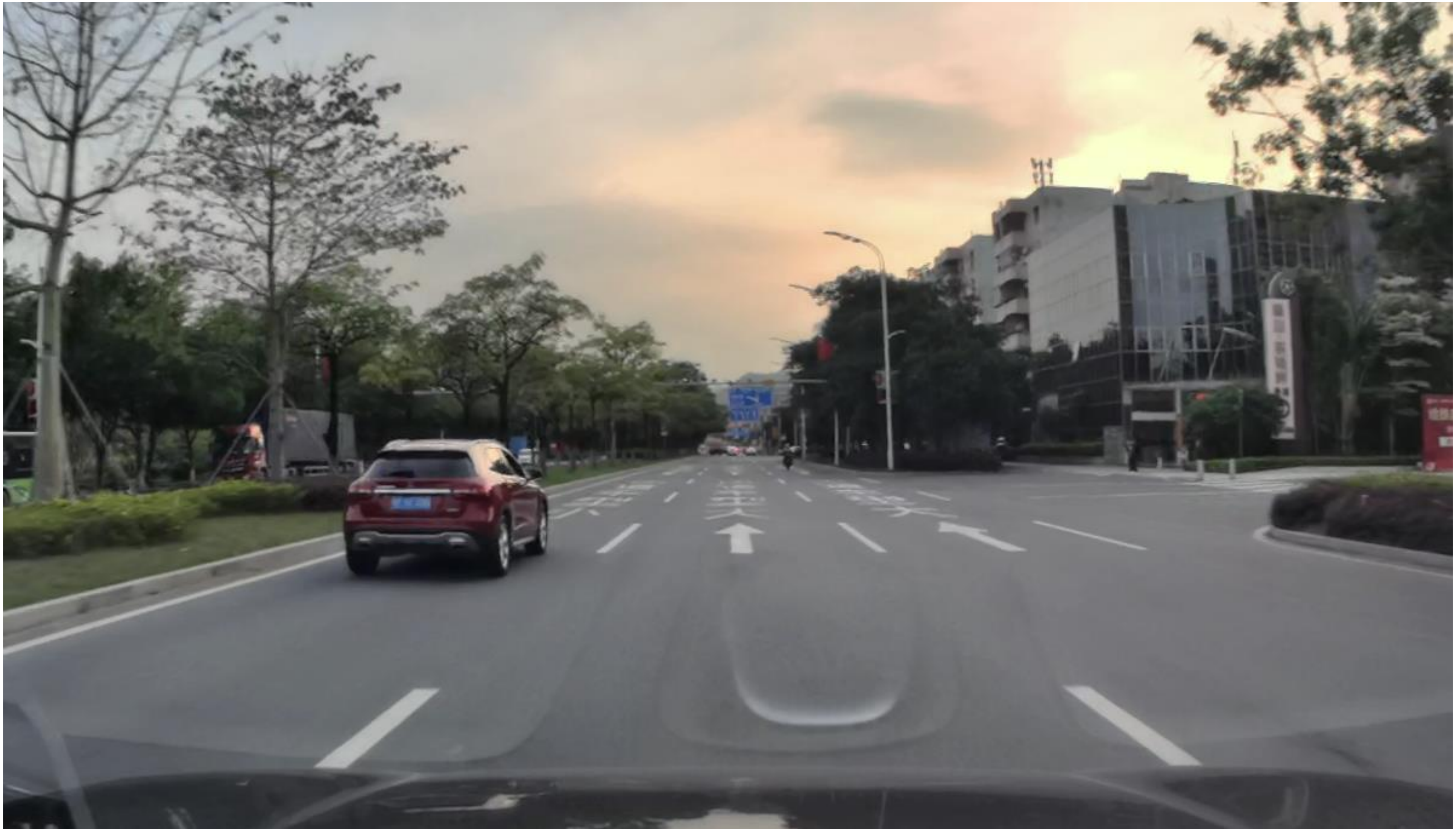

Photo for Question 13

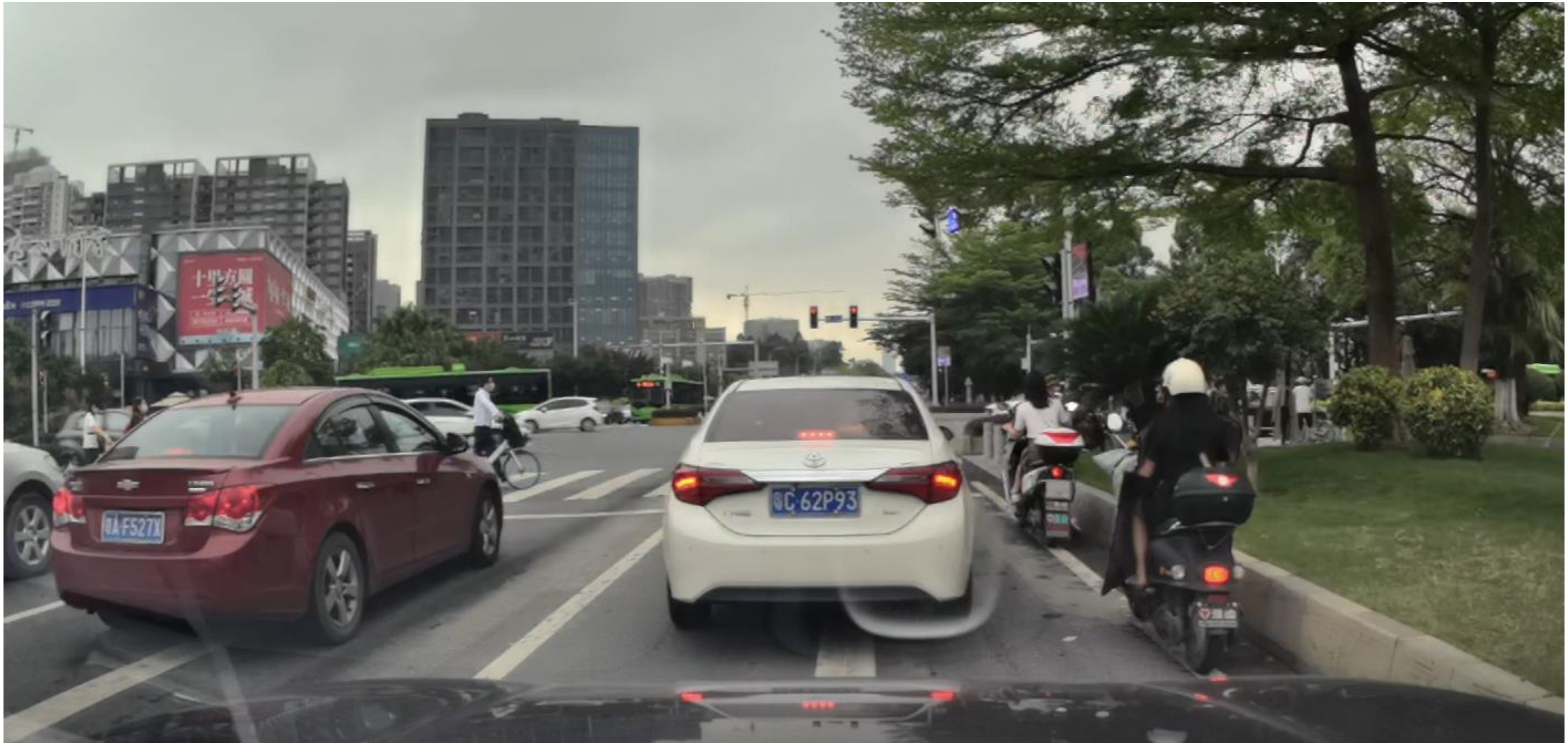

Video for Question 16

Video not provided for copyright issue.
If needed, please contact the author.

Video for Question 17

Video not provided for copyright issue.
If needed, please contact the author.

Photo for Question 18

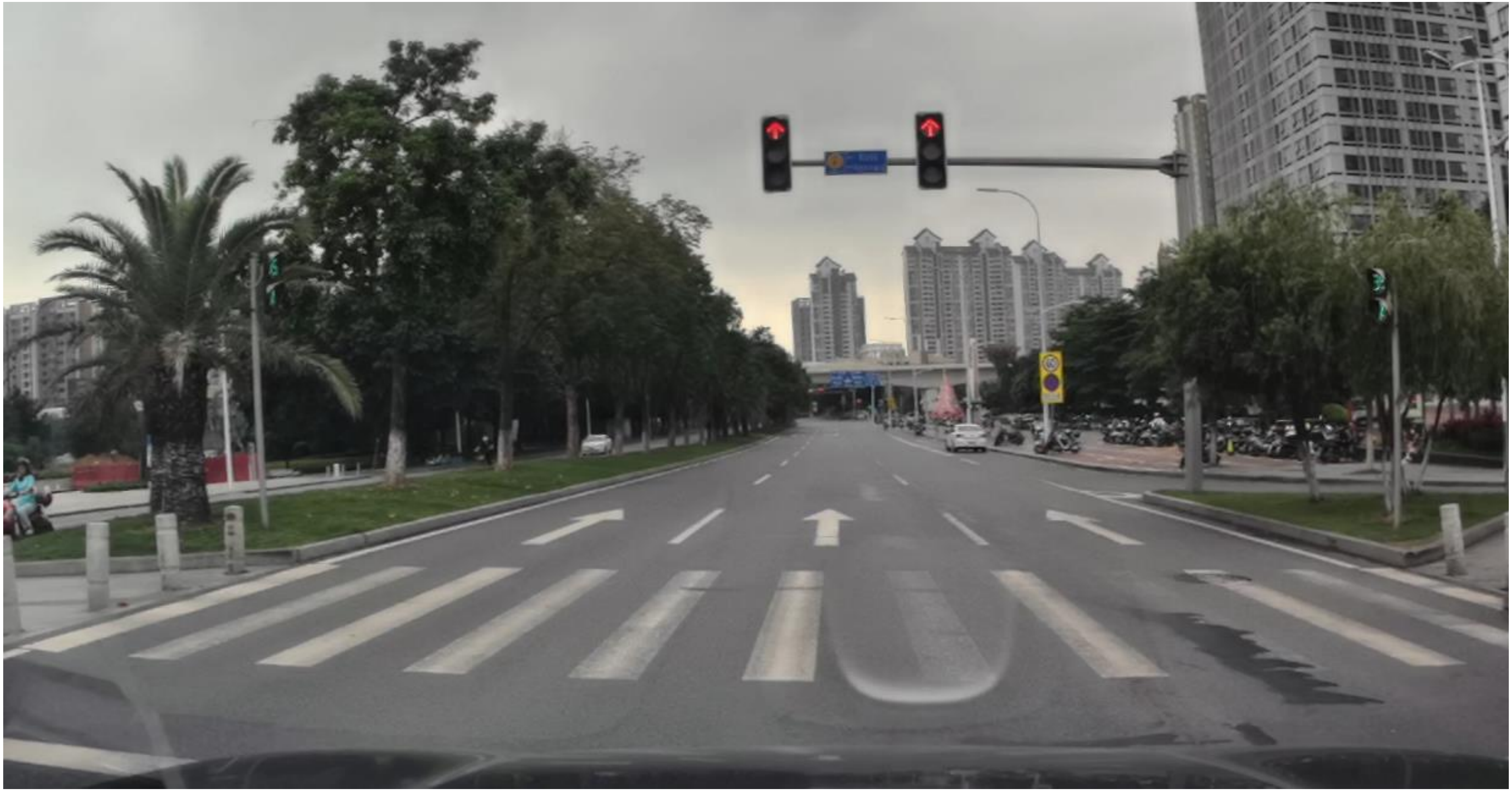

Photo for Question 21

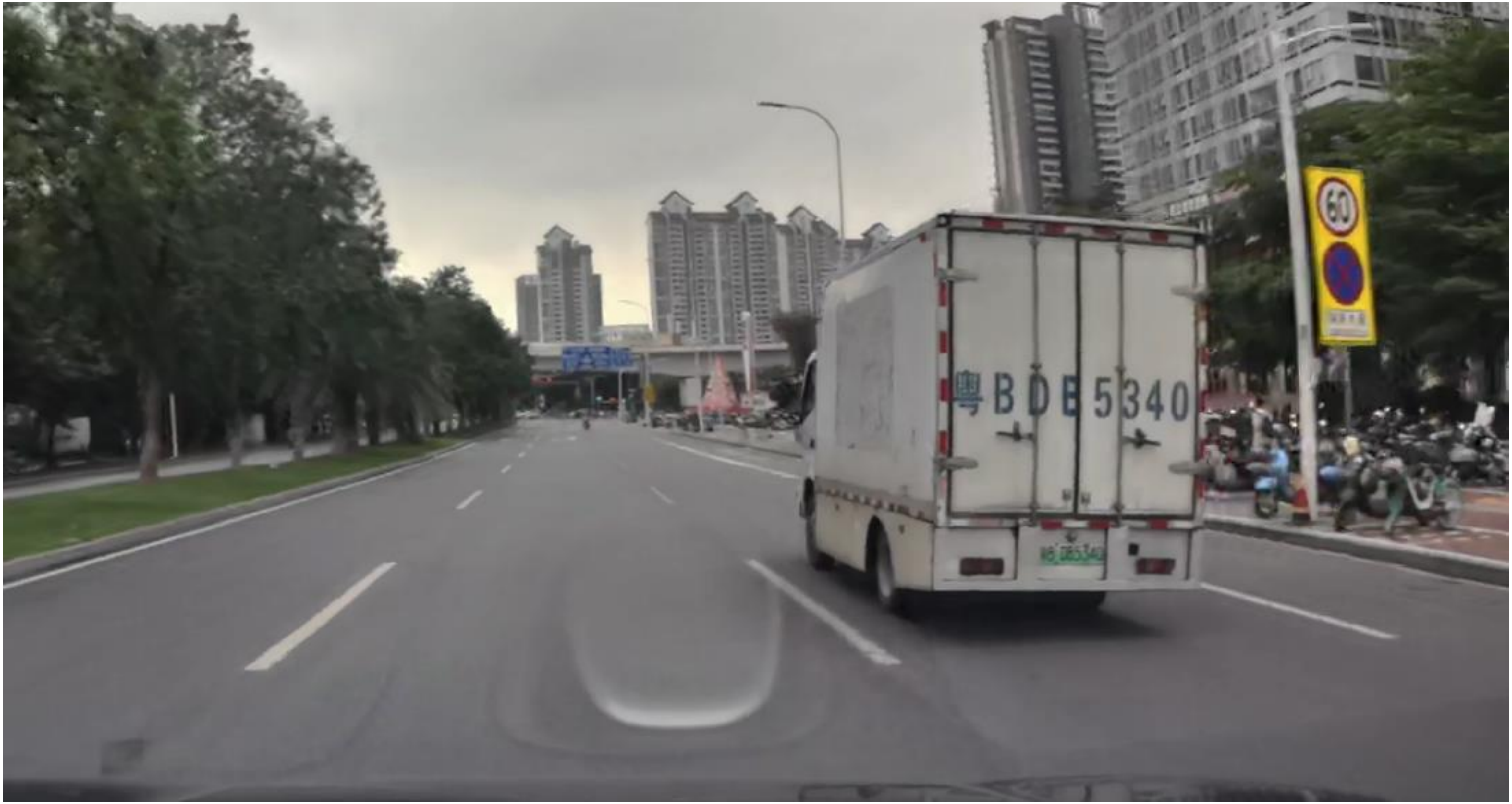

Photo for Question 22

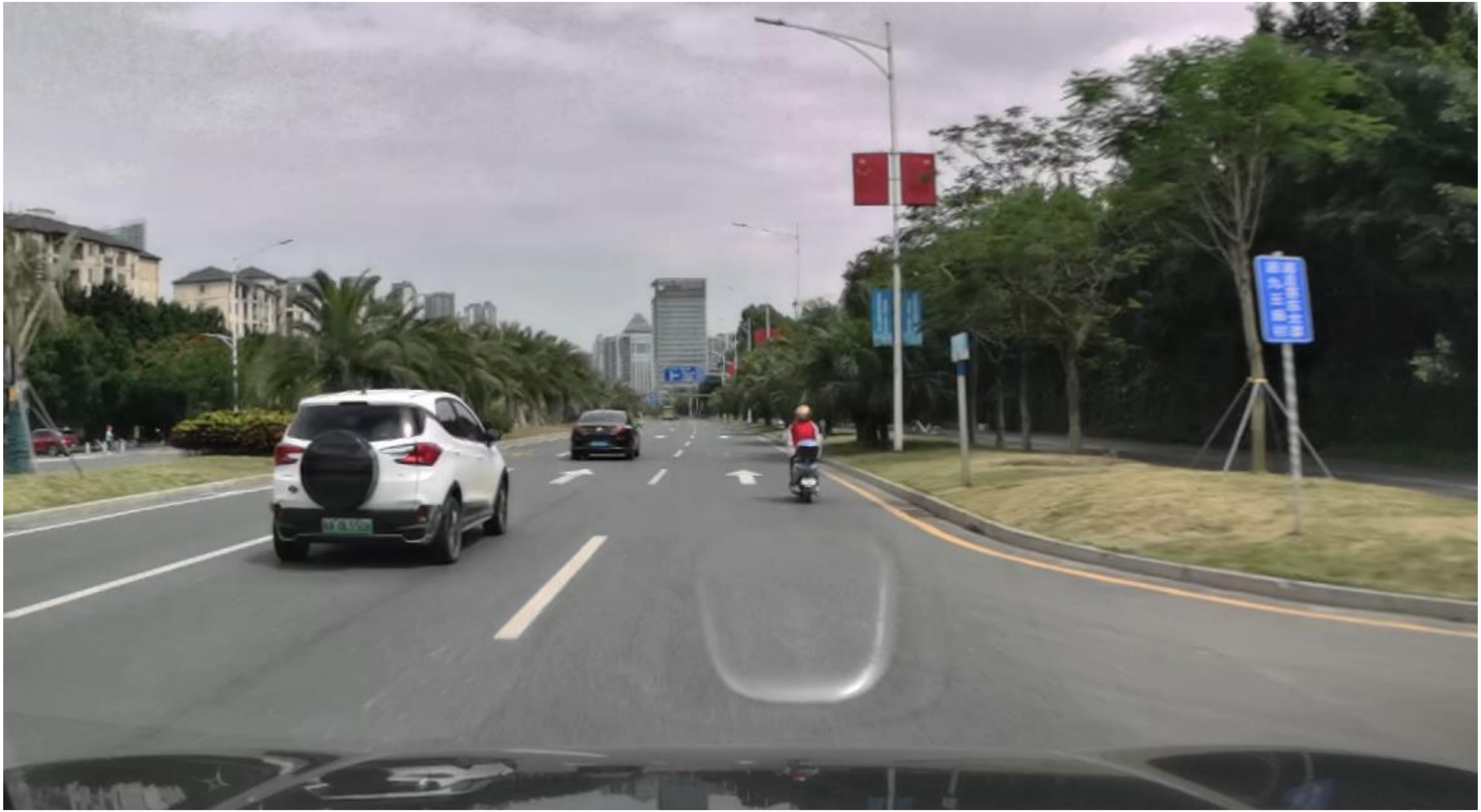

Photo for Question 36

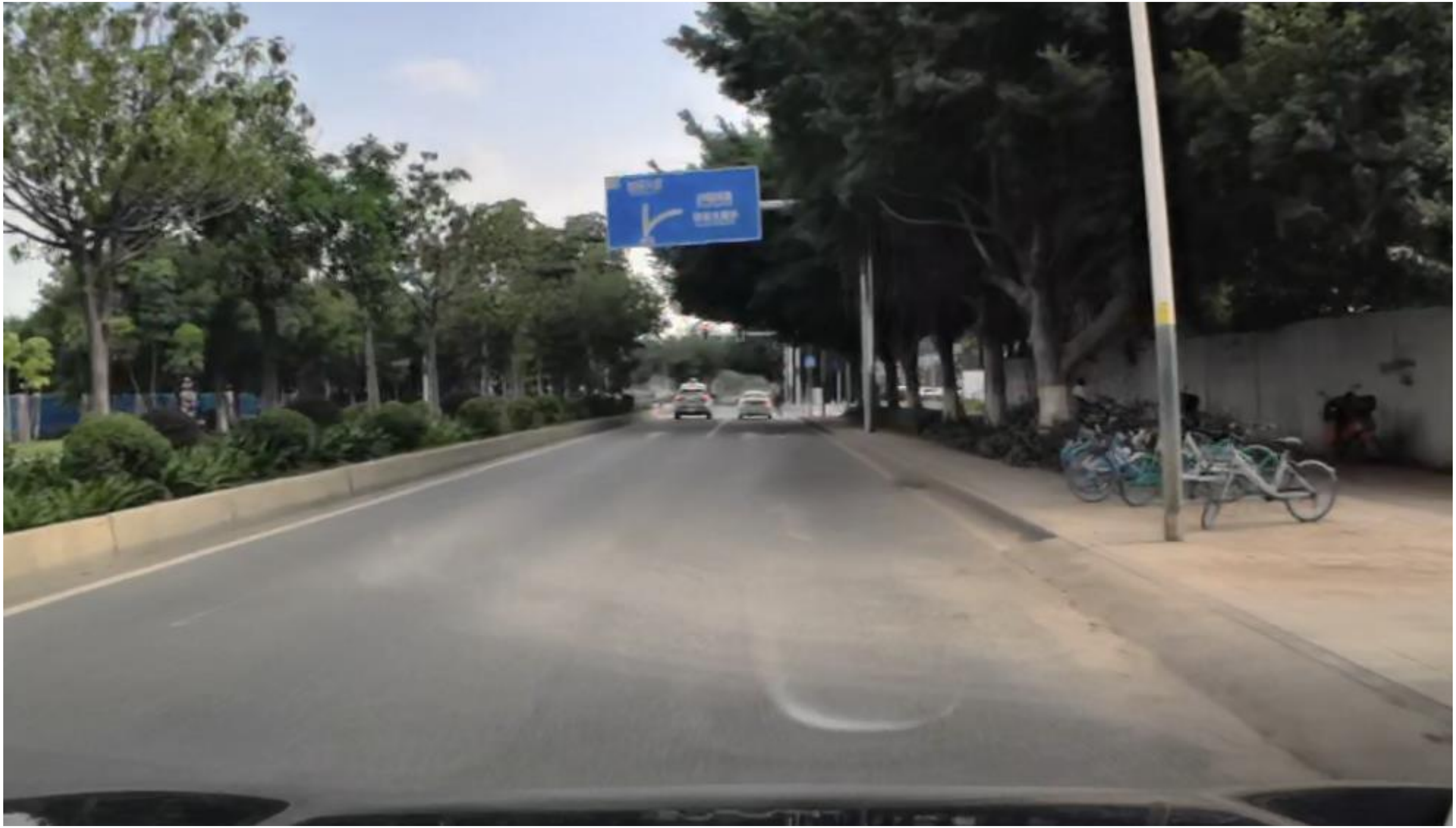

Photo for Question 37

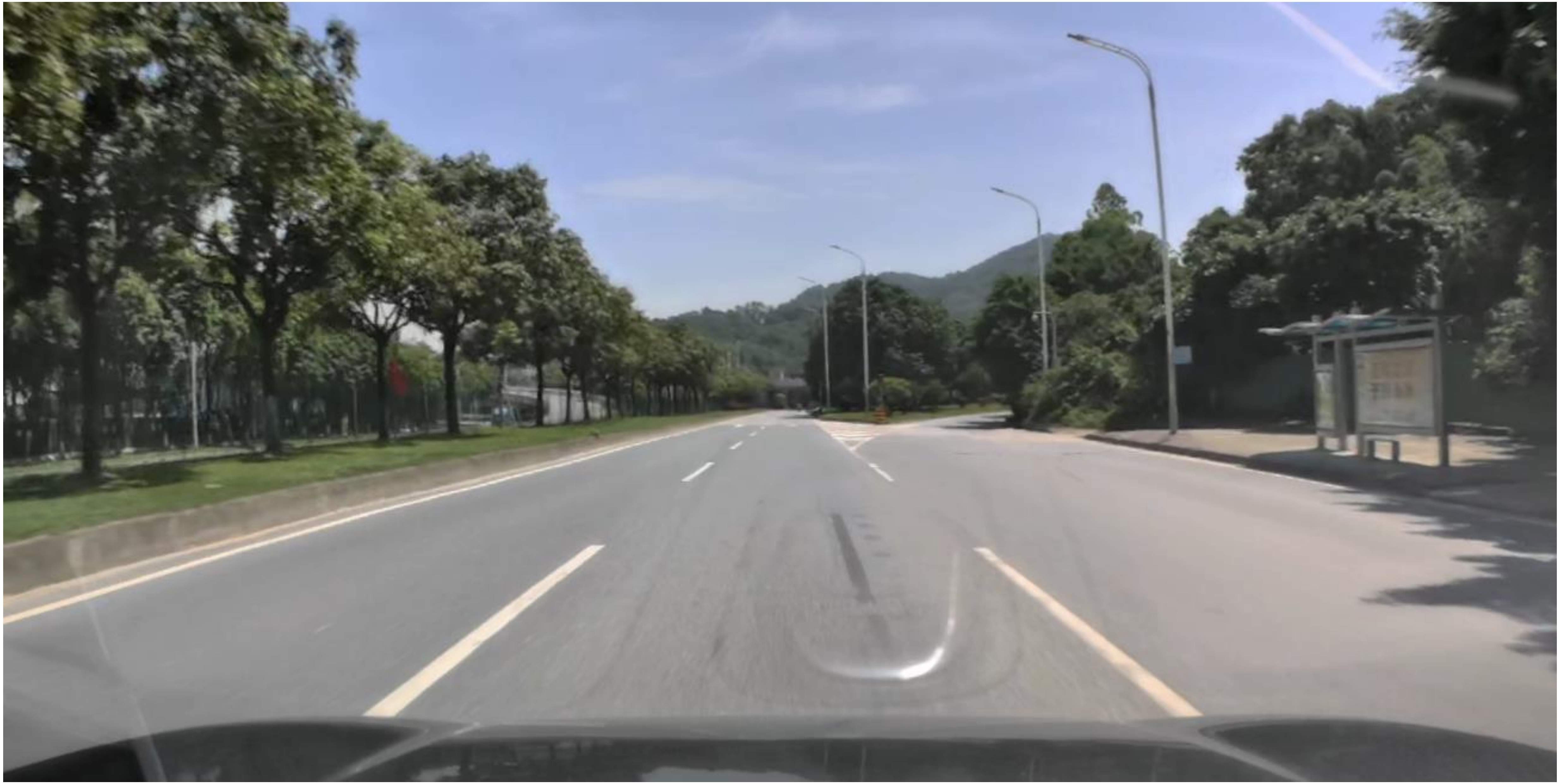

Photo for Question 41

Picture not provided for copyright issue.
If needed, please contact the author.

Photo for Question 42

Picture not provided for copyright issue.
If needed, please contact the author.

Photo for Question 44

Picture not provided for copyright issue.
If needed, please contact the author.

Photo for Question 45

Picture not provided for copyright issue.
If needed, please contact the author.

Photo for Question 46

Picture not provided for copyright issue.
If needed, please contact the author.

Photo for Question 47

Picture not provided for copyright issue.
If needed, please contact the author.

Photo for Question 48

Picture not provided for copyright issue.
If needed, please contact the author.
