# Supplementary material for: Beyond adaptive cruise control and lane centering control: drivers’ mental model of and trust in emerging ADAS technologies
Source: Front Psychol. 2023 Aug 8;14:1236062. doi: 10.3389/fpsyg.2023.1236062 (PMC10442557; doi:10.3389/fpsyg.2023.1236062)
Supplement: Supplementary file 2 [file Data_Sheet_2.docx]

# Appendix

Statements used to assess drivers’ mental models of ADAS:

| **No.** | **Statement Description** |
| --- | --- |
| 1 | As shown in the figure, when you drive in an empty lane with ADAS on, the system could keep the vehicle at a pre-set speed. |
| 2 | As shown in the figure, when you follow a lead vehicle with ADAS on, if the pre-set speed is larger than the speed of the lead vehicle, the system could keep a pre-set distance to the lead vehicle. |
| 3 | When you drive in a lane with ADAS on, the system may automatically adjust the steering wheel to keep the vehicle in the center of the lane. |
| 4 | As shown in the figure, when you drive with ADAS on, the system could react to a static object in the lane (e.g., a stopped lead vehicle due to red light). |
| 5 | As shown in the figure, when you drive with ADAS on, the system could react to the vehicle that was about to cut in at the moment indicated by the figure (i.e., the moment when the turning signal is on). |
| 6 | As shown in the figure, when you drive with ADAS on, the system could react to the vehicle that was about to cut in at the moment indicated by the figure (i.e., the moment when the wheel was over the lane mark). |
| 7 | As shown in the figure, when you drive with ADAS on, the system could react to the vehicle that was about to cut in at the moment indicated by the figure (i.e., the moment when half of the vehicle was in the ego-lane). |
| 8 | As shown in the figure, when you drive with ADAS on, the system will automatically slow down when entering a curve. |
| 9 | When you drive with ADAS on, the system will exit when you press the brake pedal. |
| 10 | As shown in the figure, when the lane marks are clear, the ADAS system in your vehicle could work on urban roads. |
| 11 | You can use the ADAS very safely on urban roads. |
| 12 | When you drive with ADAS on, the system will alert you if human intervention is required. |
| 13 | As shown in the figure, when you drive with ADAS on and stop at the traffic light, the system will automatically follow the lead vehicle when it moves again after a period of time. |
| 14 | When you drive with ADAS on, the system could react to vehicles approaching from behind. |
| 15 | When you drive with ADAS on, the system allows you to drive faster than the pre-set speed if you press the accelerator pedal. |
| 16 | As shown in the video (vehicle running on a curve with small radius), when you drive with ADAS on, the system may require you to take over. |
| 17 | As shown in the video (vehicle running on a curve with large radius), when you drive with ADAS on, the system may require you to take over. |
| 18 | As shown in the figure, when you drive with ADAS on, the system can recognize and react to traffic lights. |
| 19 | When you drive with ADAS on, in some cases, the system can slow down to a complete stop. |
| 20 | With clear lane markings, you can activate the ADAS at any speed (e.g., below 30 km/h). |
| 21 | As shown in the figure, when you drive with ADAS on, the system can recognize road traffic signs (e.g., speed limit signs) and automatically adjust the set speed accordingly. |
| 22 | As shown in the figure, when you drive with ADAS on, the system can react to a motorcycle or electric bicycle driving in front of the vehicle in the ego-lane. |
| 23 | When you drive with ADAS on, if the speed exceeds the current road speed limit, the system will alert you. |
| 24 | When you drive with ADAS on, if the vehicle in front is slow, the system can automatically change lanes. |
| 25 | When you drive with ADAS on, the system will automatically change lanes if there is a stationary object in front of the lane (e.g., a parked vehicle). |
| 26 | When you drive with ADAS on, the vehicle will not react to your action of turning the steering wheel. |
| 27 | When you drive with ADAS on, the system will exit if you manually turn on the turn signal. |
| 28 | When you drive with ADAS on, the system will follow your acceleration command when you press the accelerator pedal, but when you release the accelerator pedal, the system will return to a pre-set speed. |
| 29 | When you drive with ADAS on, you can directly control the direction of the vehicle if you manually turn the steering wheel. |
| 30 | When you drive with ADAS on, if your eyes are off the road for an extended period of time, the system will alert you. |
| 31 | When you drive with ADAS on, the system will alert you if your hands are off the wheel for an extended period of time. |
| 32 | When you drive with ADAS on, if your eyes are off the road for an extended period of time and you ignore the system alarms (if any), the system will exit. |
| 33 | When you drive with ADAS on, if your hands are off the wheel for an extended period of time and you ignore the system alarms (if any), the system will exit. |
| 34 | When you drive with ADAS on, if your eyes are off the road for an extended period of time and you ignore the system alarms (if any), the system will stop the vehicle and turn on the hazard lights. |
| 35 | When you drive with ADAS on, if your hands are off the wheel for an extended period of time and you ignore the system alarms (if any), the system will stop the vehicle and turn on the hazard lights. |
| 36 | As shown in the figure, when you drive with ADAS on, if the lane markings on the road are unclear or there are no lane markings, the system may have difficulties. |
| 37 | As shown in the figure, when you drive with ADAS on, the system may have difficulties when ego lane divides (e.g., highway exit) or multiple lane merge (e.g., ramp joining the highway). |
| 37 | When you drive with ADAS on, if the lane markings are not clear, the ADAS may have difficulties entering or exiting the highway. |
| 39 | When you drive with ADAS on, if the traffic is heavy, the system may have difficulties entering or exiting the highway. |
| 40 | When you drive with ADAS on, the system may have difficulties when the on-board sensors (such as cameras, radar, etc.) are dirty or blocked. |
| 41 | As shown in the figure, when you drive with ADAS on, the system may have difficulties driving in poor weather conditions (e.g., heavy rain, heavy snow, fog). |
| 42 | As shown in the figure, when you drive with ADAS on, the system may have difficulties when the roads are slippery (e.g., wet ground and icy ground). |
| 43 | When you drive with ADAS on, the system may have difficulties when the signal of satellite-based navigation systems (e.g., GPS) is weak. |
| 44 | As shown in the figure, when you drive with ADAS on, the system may have difficulties driving through the tunnel. |
| 45 | As shown in the figure, when you drive with ADAS on, the system may have difficulties when there are reflections on the road surface (e.g., light from the sun). |
| 46 | As shown in the figure, when you drive with ADAS on, the system may have difficulties when there are only lane markings on one side of the road. |
| 47 | As shown in the figure, when you drive with ADAS on, the system may have difficulties when there is a construction area on the road. |
| 48 | As shown in the figure, when you drive with ADAS on, the system may have difficulties when there are traffic cones on the road. |
| 49 | When you drive with ADAS on, the system may have difficulties distinguishing between solid and dashed lane markings on the road. |
